# Actin cytoskeleton self-organization in single epithelial cells and fibroblasts under isotropic confinement

**DOI:** 10.1242/jcs.220780

**Published:** 2019-03-07

**Authors:** Salma Jalal, Shidong Shi, Vidhyalakshmi Acharya, Ruby Yun-Ju Huang, Virgile Viasnoff, Alexander D. Bershadsky, Yee Han Tee

**Affiliations:** 1 Mechanobiology Institute, National University of Singapore, Singapore 117411; 2 Cancer Science Institute of Singapore, National University of Singapore, Singapore 117599; 3 Department of Obstetrics & Gynaecology, National University Hospital, Singapore 119228; 4 Department of Anatomy, Yong Loo Lin School of Medicine, National University of Singapore, Singapore 117599; 5 Centre National Pour la Recherche Scientifique, Singapore 117411; 6 Department of Biological Sciences, National University of Singapore, Singapore 117558; 7 Department of Molecular Cell Biology, Weizmann Institute of Science, Rehovot 7610001, Israel

**Keywords:** Actin cytoskeleton, Self-organization, Epithelial cell, Fibroblast, Epithelial–mesenchymal transition

## Abstract

Actin cytoskeleton self-organization in two cell types, fibroblasts and epitheliocytes, was studied in cells confined to isotropic adhesive islands. In fibroblasts plated onto islands of optimal size, an initially circular actin pattern evolves into a radial pattern of actin bundles that undergo asymmetric chiral swirling before finally producing parallel linear stress fibers. Epitheliocytes, however, did not exhibit succession through all the actin patterns described above. Upon confinement, the actin cytoskeleton in non-keratinocyte epitheliocytes was arrested at the circular stage, while in keratinocytes it progressed as far as the radial pattern but still could not break symmetry. Epithelial–mesenchymal transition pushed actin cytoskeleton development from circular towards radial patterns but remained insufficient to cause chirality. Knockout of cytokeratins also did not promote actin chirality development in keratinocytes. Left–right asymmetric cytoskeleton swirling could, however, be induced in keratinocytes by treatment with small doses of the G-actin sequestering drug, latrunculin A in a transcription-independent manner. Both the nucleus and the cytokeratin network followed the induced chiral swirling. Development of chirality in keratinocytes was controlled by DIAPH1 (mDia1) and VASP, proteins involved in regulation of actin polymerization.

This article has an associated First Person interview with the first author of the paper.

## Abstract (precis)

**Summary:** Epitheliocytes cannot develop the F-actin patterns typically observed in fibroblasts, but can do so after treatments affecting actin polymerization. Regulators of actin polymerization, DIAPH1 and VASP, control this process.

## INTRODUCTION

Differences in the morphogenetic behavior between fibroblasts and epithelial cells have been well documented. *In vivo*, epithelial cells possess apico-basal polarity and are organized into sheets of cells that are connected to each other via E-cadherin-mediated cell–cell junctions and attached to the basement membrane via integrin-mediated adhesions. In contrast, fibroblasts are embedded in and attached to the extracellular matrix of connective tissue and do not readily adhere to neighboring cells. The difference between morphology and migratory characteristics of epithelial cells and fibroblasts are accompanied by differences in their actin cytoskeleton organization. The most prominent element of the actin cytoskeleton in epithelial cells is the adhesion belt associated with the cell–cell junctions and located at the apical zone of the cells (Braga, 2016; Volberg et al., 1986; Zhang et al., 2005). In typical fibroblasts, the actin cytoskeleton is organized into parallel linear actomyosin bundles (stress fibers) associated with cell–matrix adhesions (focal adhesions) (Burridge, 2017; Geiger, 1979; Heath and Dunn, 1978). Consistently, upon epithelial–mesenchymal transition (EMT) the circumferential bundles in epithelial cells are succeeded by a system of parallel stress fibers (Boyer et al., 1989; Haynes et al., 2011). Actin dynamics and several actin regulatory proteins have been shown to be involved in EMT (Gardberg et al., 2013; Li et al., 2010; Mori et al., 2009; Shankar and Nabi, 2015).

It is well documented that outside-in signaling from adhesion receptors directs the formation of the actin cytoskeleton within cells (Braga, 2016; Geiger et al., 2009). Therefore, it is possible that differences in actin cytoskeleton self-organization between epithelial cells and fibroblasts are determined by the differences in available extracellular adhesions (cell–cell and cell–matrix versus cell–matrix only). On the other hand, it is also possible that the actin cytoskeleton itself has intrinsically different morphogenetic potentials in these two cell types. To reveal the difference in the morphogenetic potential of the actin cytoskeleton independently of differences in adhesion conditions, we study the self-organization of the actin cytoskeleton in a standardized microenvironment provided by microfabricated adhesive patterns (Théry, 2010; Théry et al., 2006). Under such conditions, cells are deprived of adhesions to neighboring cells and only contact extracellular matrix proteins (specifically fibronectin or collagen). To dissect the processes of actin cytoskeleton self-organization from potential interplay with cell shape changes during migration, we confined cells to isotropic circular adhesive islands so that they acquire discoid morphology and do not change shape.

We have previously used this system of isotropic confinement to investigate the process of actin cytoskeleton self-organization that leads to formation of parallel linear stress fibers in isolated fibroblasts (Tee et al., 2015). This setting revealed unique patterns of actin development with initially symmetric circular patterns consisting of circumferential bundles that progressed into radial patterns composed of bundles of actin anchored at focal adhesions that grow radially inwards from the cell periphery. These patterns were followed by a symmetry-breaking process that produced a novel chiral pattern in which all radial bundles of actin tilted in one direction. This tilting converts the centripetal actin flow into a swirling motion that persists until arrays of parallel stress fibers are formed (the linear pattern).

We systematically compared actin cytoskeleton self-organization between epithelial cells and fibroblasts, as well as between different types of epithelial cells. We found that the morphogenetic repertoire of actin cytoskeleton patterns in epithelial cells was limited to the circular actin pattern in non-keratinocyte epitheliocytes and extended to the radial actin pattern in keratinocytes, but did not include the chiral actin pattern present in fibroblasts.

The presence of the cytokeratin network in epithelial cells, its depletion during EMT and absence in fibroblasts, makes its interplay with the actin cytoskeleton a possible candidate to explain the differences in the actin cytoskeleton development between the two cell types. However, we demonstrated that the pattern of actin cytoskeleton self-organization in keratinocytes does not require cytokeratin filaments. Growth factor-induced EMT pushed development of the actin cytoskeleton in some epithelial cells from circular towards radial actin patterns but was insufficient to induce chiral actin pattern development.

We found, however, that chiral actin pattern development can be induced in keratinocytes by treatment with low doses of the G-actin sequestering drug latrunculin A. Both nuclei and the cytokeratin network followed the chiral actin swirling without impeding it. The induction of chiral actin patterns was independent of transcriptional changes but involved at least two major regulators of actin polymerization, the formin DIAPH1 (also known as mDia1) and VASP.

## RESULTS

### Actin cytoskeleton self-organization in isolated fibroblasts depends on projected cell area

**Fig. 1.**
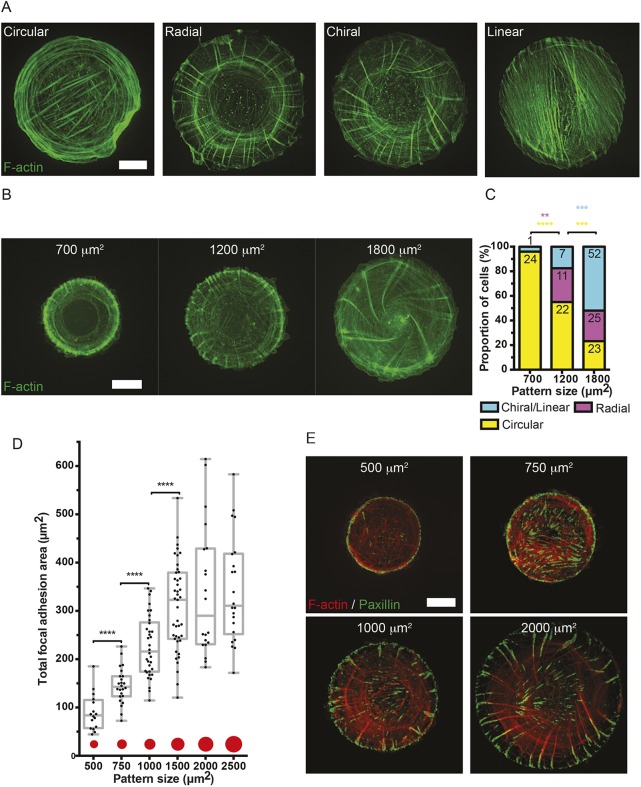
**Evolution of actin cytoskeleton self-organization in fibroblasts confined to adhesive islands of various sizes.** (A) Representative images showing F-actin (phalloidin staining) distribution in HFFs fixed 6 h after seeding on fibronectin-coated islands of 1800 µm^2^. Cells normally initially organize a circular pattern consisting of circumferential actin bundles. After ∼3 h, the circular pattern is succeeded by the radial actin pattern upon radially symmetric growth of actin bundles (radial fibers, RFs) inwards from the cell periphery. Simultaneously, circumferential actin bundles parallel to the cell edge (transverse fibers, TFs) move centripetally along RFs. After ∼6 h, in a majority of cells, all RFs spontaneously tilt in one direction to form the chiral actin pattern, which persists until actin stress fibers ultimately linearize in the linear actin pattern. See Movie 1. Scale bar: 10 µm. (B) Representative frames showing actin pattern development in HFFs expressing LifeAct–GFP seeded on 700, 1200 or 1800 µm^2^ fibronectin-coated islands and imaged overnight. Scale bar: 10 µm. (C) Bar chart showing fractions of cells with actin cytoskeleton demonstrating circular, radial, chiral or linear pattern by the end of filming (8–12 h) on each island size imaged under conditions as in B. Numbers of cells displaying each actin pattern are labeled on top of each bar per condition, pooled from at least three independent experiments. Fisher's exact tests were used to assess significance between the fractions of cells displaying circular (yellow asterisk), radial (magenta asterisk) or chiral/linear (cyan asterisk) actin pattern in pairs of adhesive island sizes (700 vs 1200, 1200 vs 1800 µm^2^); **P<0.01, ***P<0.001, ****P<0.0001. (D) Box-and-whiskers plot showing the total focal adhesion area per cell in cells imaged under conditions of E. The total focal adhesion area was measured in 16, 24, 34, 43, 20 and 22 cells on island sizes of 500, 750, 1000, 1500, 2000 and 2500 µm^2^, respectively. Mann–Whitney tests were used to assess significance between the total focal adhesion areas in pairs of island sizes; ****P<0.0001. The box represents the 25–75th percentiles, and the median is indicated. The whiskers show the complete range from minimum to maximum values. Points superimposed on the graph show all values. (E) Representative images showing focal adhesion (paxillin immunostaining) and F-actin (phalloidin staining) distribution in fibroblasts fixed 8 h after seeding on fibronectin-coated islands of different sizes. Scale bar: 10 µm.

In isolated human foreskin fibroblasts (HFFs) confined to circular adhesive islands of 1800 µm^2^ in area covered with fibronectin, the actin cytoskeleton initially self-organizes into a circular pattern of circumferential actin bundles with some lamellipodial protrusions (Fig. 1A). Sometimes these cells demonstrate sparse ventral stress fibers that are not organized into arrays (Movie 1). Within a few hours this circular pattern is succeeded by a symmetric radial pattern formed by radial fibers (RFs) that grow inwards from focal adhesions, and transverse fibers (TFs) oriented orthogonally to the RFs and parallel to the cell edge (Fig. 1A). After some time, a symmetry-breaking event occurs and all RFs tilt in the counter-clockwise direction forming the chiral pattern, before actin bundles eventually organize into the linear pattern containing parallel linear stress fibers (Fig. 1A) similar to those seen in fibroblasts polarized on planar fibronectin-coated substrates (Fig. S1A, upper frame). This development and progression through circular to linear actin patterns is consistent with previously published data (Tee et al., 2015).

Given that different cell types have differential capacities to spread on planar adhesive surfaces (coated with fibronectin or collagen, Fig. S1B), adhesive islands of different sizes (generated by either microcontact printing or stenciling, see Materials and Methods and Fig. S1C) were used to systematically assess whether the projected cell area affects actin cytoskeleton self-organization.

When HFFs expressing LifeAct–GFP were confined to small adhesive islands (700 µm^2^ in area), actin cytoskeleton development was restricted to circular patterns in 24 out of 25 cells imaged overnight (Fig. 1B, represented by the yellow bar in Fig. 1C). On medium-sized adhesive islands (1200 µm^2^ in area), the circular pattern (yellow bar in Fig. 1C) was still demonstrated by more than 50% of cells, but the remaining fraction of cells demonstrated progression to the radial (Fig. 1B, magenta bar in Fig. 1C) and chiral/linear (cyan bar in Fig. 1C) actin patterns. Finally, on islands of 1800 µm^2^ the fraction of cells demonstrating the circular pattern was less than a quarter, while the majority of cells were able to develop the chiral pattern (Fig. 1B,C).

Transition from the circular to radial patterns of actin involves the growth of RFs anchored at focal adhesions. To further probe how spread area affects actin cytoskeleton self-organization, we examined its effects on focal adhesions as visualized by paxillin immunostaining (Fig. 1D,E). Total focal adhesion area in fibroblasts confined to adhesive islands increased with projected cell area. There was a strong statistically significant increase of total focal adhesion area in cells on patterns with areas of 500, 750, 1000 and 1500 µm^2^ (Fig. 1D). Of note, the increase in total focal adhesion area was somewhat greater than the increase in projected cell area. In particular, there is a mild statistically significant increase between the total focal adhesion area normalized to the projected cell area between patterns with areas of 500 and 1000 µm^2^ (Fig. S1D). The increase in total focal adhesion area approached a plateau at the island size of 1500 µm^2^, so that the total focal adhesion area in cells on islands of larger sizes (2000 and 2500 µm^2^) remained constant (Fig. 1D), while normalized total focal adhesion area decreased (Fig. S1D).

### Non-keratinocyte epithelial cells confined to circular adhesive islands demonstrate only the initial stages of actin cytoskeleton self-organization

**Fig. 2.**
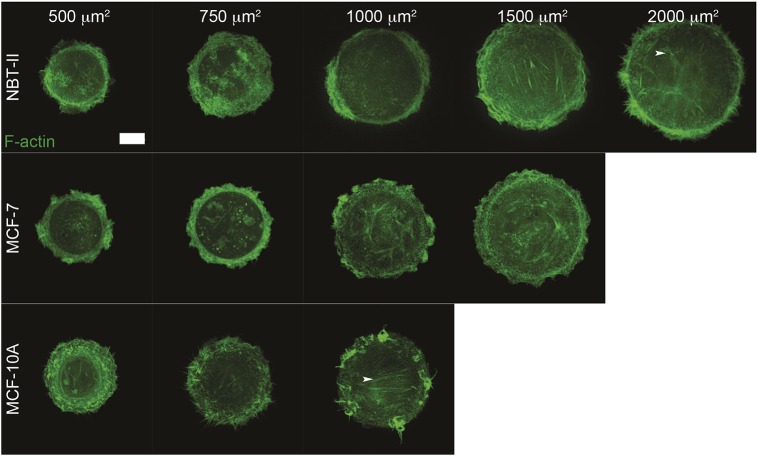
**Actin cytoskeleton pattern development in non-keratinocyte epithelial cells confined to adhesive islands of various sizes.** Representative frames of single attached epithelial cells expressing LifeAct–GFP (to visualize F-actin) that were seeded onto substrates evenly micropatterned with adhesive islands of different sizes (500, 750, 1000, 1500, 2000 and 2500 µm^2^, see Materials and Methods and Fig. S1C) and imaged overnight. MCF-10A and MCF-7 cells were seeded on collagen-coated substrates while NBT-II cells were seeded on fibronectin-coated substrates. Blank frames correspond to situations when cells did not fill circular islands of the specified size (see Table S1). White arrowheads label sparse non-organized ventral stress fibers. Scale bar: 10 µm.

To examine actin cytoskeleton self-organization in epithelial cells confined to circular adhesive islands, a selection of cell lines from different tissues of origin (including mammary, bladder and skin) was used. Epithelial cell morphology in standard culture is different from fibroblasts, in that cells do not elongate and remain discoid (Fig. S1A, lower frame). Of note, the spreading capacity of these lines (MCF-7, MCF-10A and NBT-II) on planar matrix-coated substrates is much lower than that of fibroblasts (Fig. S1B). All non-keratinocyte epithelial cells tested preferentially filled smaller adhesive islands and did not fill larger pattern sizes well (Table S1). In epithelial cells not originating from skin, imaged overnight or fixed 20–24 h after seeding, actin cytoskeleton development was mostly restricted to the circular pattern characterized by circumferential actin bundles and protrusive membrane activity at the cell periphery (Fig. 2; Fig. S2). Sparse non-organized ventral stress fibers sometimes observed in these cells (white arrowheads, Fig. 2) were usually more dynamic than ventral stress fibers in fibroblasts (Movie 2 compared to Movie 1).

Next, we investigated how the actin cytoskeleton in epithelial cells changes during EMT and, in particular, whether the actin pattern of self-organization typical for fibroblasts can be reproduced in epithelial cells undergoing EMT. Epidermal growth factor (EGF) induction of EMT in rat bladder carcinoma cells (NBT-II) was used as a model system (Boyer et al., 1997). In a colony assay, EGF treatment induced loosening and partial scattering of NBT-II cell colonies (Fig. S3A). In agreement with previous studies, EMT was accompanied by upregulation of Slug (also known as SNAI2) and downregulation of E-cadherin (Fig. S3B).

**Fig. 3.**
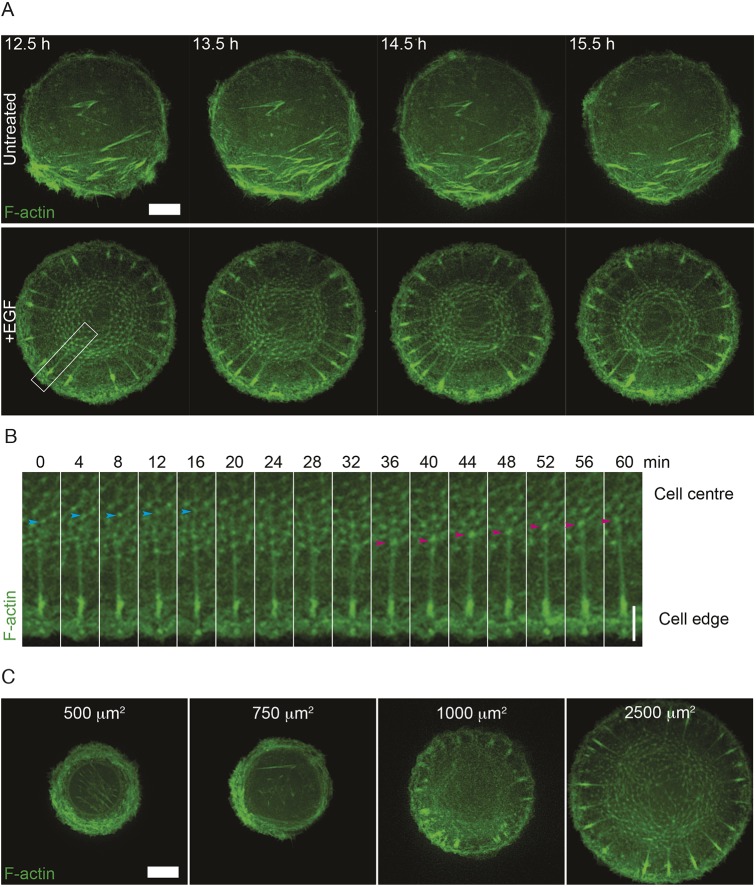
**Non-keratinocyte NBT-II epithelial cells acquire the ability to form radial actin patterns after EMT.** (A) Representative time-lapse series showing actin cytoskeleton in control untreated NBT-II cells expressing LifeAct–GFP and seeded on 1800 µm^2^ fibronectin-coated islands (upper panel), and NBT-II cells pretreated with 100 ng ml^−1^ EGF for 3 days and replated on 1800 µm^2^ fibronectin-coated islands in the continued presence of EGF (lower panel). Time in hours after seeding is indicated in the upper left corner of frames; the first frame corresponds to the time when untreated cells filled the entire area of the adhesive circular island. Frames for treated cells are shown at corresponding time points after seeding. See Movies 2 and 3. Scale bar: 10 µm. (B) Montage over 1 hour taken from region highlighted by a white box shown in the first frame of the EGF-treated cell from A. The cyan arrowhead follows an aster from the start of the time series until it disappears from view, while the magenta arrowhead follows an aster that appears at 36 min and persists until the end of the series. See Movie 4. Scale bar: 5 µm. (C) Representative frames of actin pattern formation in NBT-II cells replated on fibronectin-coated islands of different sizes after 3 days of treatment with 100 ng ml^−1^ EGF. Cells were transfected with LifeAct–GFP 1 day before replating and imaging overnight. Scale bar: 10 µm.

Wild-type untreated NBT-II cells spread on fibronectin-coated islands mainly demonstrated circumferential bundles of actin and membrane protrusions along with some sparse ventral stress fibers (Figs 2 and 3A, upper panel; Movie 2). After treatment with EGF for at least 1 day to induce the EMT phenotype, the actin cytoskeleton is able to self-organize into a radial actin pattern (Fig. 3A, lower panel; Movie 3 and Fig. S3C). This pattern contains RFs originating from focal adhesion-like structures resembling those in fibroblasts (Fig. 3A, lower panel, compared to Fig. 1A). Unlike the radial actin pattern in fibroblasts, the RFs in EGF-treated NBT-II cells are connected to a centrally located dynamic actin array consisting of numerous actin foci connected by thin actin bundles (Fig. 3A, lower panel). This array appeared to assemble at the periphery and disassemble in the central area of the cell, thus demonstrating a centripetal flow (Fig. 3B; Movies 3 and 4). Similar to what was seen in fibroblasts, the self-organization of actin into radial patterns in EGF-treated NBT-II cells showed size dependence (Fig. 3C). Only 2 out of 22 cells imaged overnight confined to small (500 and 750 µm^2^) adhesive islands developed RFs, while 3 out of 15 cells imaged on medium-sized islands (1000 µm^2^) and 6 out of 16 treated cells imaged overnight on large islands (1500, 2000 and 2500 µm^2^) were able to develop RFs (Fig. 3C).

To further examine the effect of EMT on the morphogenetic potential of the actin cytoskeleton in epithelial cells confined to adhesive islands, we studied a series of ovarian cancer cell lines with different EMT status (Nieto et al., 2016; Tan et al., 2014). We used four cell lines that differed in their EMT score (Fig. S4A). The EMT score is based on transcriptomic analysis of 218 genes (epithelial: 170, mesenchymal: 48), and ranges from −1 for the most epithelial phenotype up to +1 for the most mesenchymal phenotype (Nieto et al., 2016; Tan et al., 2014). Similar to other non-keratinocyte epithelial cell lines tested, the ovarian cancer cell line with epithelial phenotype (PEO1, Fig. S4B) only developed circular actin patterns (Fig. S4C) and generally did not fill the entire area of large adhesive islands (Table S2). In contrast, HEYA8 cells with the most mesenchymal phenotype (Fig. S4B) demonstrated radial or linear actin patterns in the majority of imaged cells (Fig. S4C,D). The cell lines with intermediate EMT scores (intermediate epithelial, OVCA429 and intermediate mesenchymal, SKOV3) were able to cover the entire surface of large adhesive islands and sometimes formed RFs albeit with lower frequency than the mesenchymal type cell line, HEYA8. On large adhesive islands (1500, 2000 and 2500 µm^2^), 6 out of 26 imaged OVCA429 and 3 out of 47 imaged SKOV3 were able to develop RFs compared to 29 out of 39 for HEYA8 (where seven of those cells demonstrated transition to the linear pattern, Fig. S4D). Similar to fibroblasts, on small adhesive islands (500, 750 and 1000 µm^2^) all of these cells were predominantly circular.

### The actin cytoskeleton in keratinocytes self-organizes into a radial actin pattern, which can develop left–right asymmetry upon treatment with low doses of latrunculin A

**Fig. 4.**
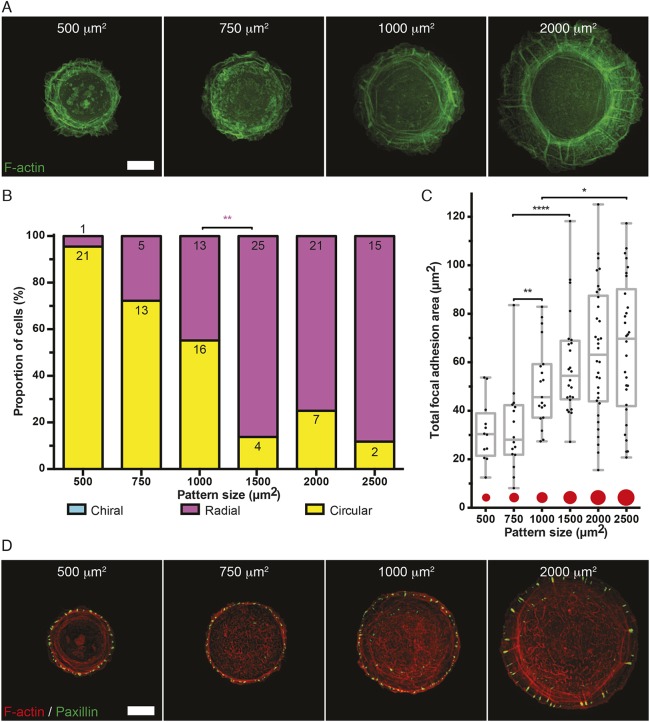
**Actin cytoskeleton self-organization in keratinocytes confined to adhesive islands of various sizes.** (A) Representative frames showing actin patterns in keratinocytes (HaCaT) expressing LifeAct–GFP or mRuby–Lifeact (pseudo-colored green) seeded on fibronectin-coated islands of different sizes and imaged overnight. Scale bar: 10 µm. (B) Bar chart showing fractions of cells with actin cytoskeletons demonstrating the circular, radial or chiral pattern by the end of filming (6–12 h) on each island size (500, 750, 1000, 1500, 2000 and 2500 µm^2^). Numbers of cells displaying each actin pattern are labeled on top of each bar per condition, pooled from six independent experiments. Fisher's exact tests were used to assess significance between the fractions of cells displaying radial (magenta asterisk) actin pattern in pairs of adhesive island sizes (500 vs 750, 750 vs 1000, 1000 vs 1500, 1500 vs 2000, 2000 vs 2500 µm^2^); **P<0.01. (C) Box-and-whiskers plot showing the total focal adhesion area per cell from cells imaged under conditions of D. The total focal adhesion areas were measured in 12, 16, 19, 24, 34 and 30 cells pooled from two independent experiments plated on islands of 500, 750, 1000, 1500, 2000 and 2500 µm^2^ in area, respectively. Mann–Whitney tests were used to assess significance between the total focal adhesion areas in pairs of island sizes; *P<0.05, **P<0.01, ****P<0.0001. The box represents the 25–75th percentiles, and the median is indicated. The whiskers show the complete range from minimum to maximum values. Points superimposed on the graph show all values. (D) Representative images showing focal adhesion (paxillin immunostaining) and F-actin (phalloidin staining) distribution in HaCaT cells fixed and stained 24 h after seeding on fibronectin-coated islands of different sizes. Scale bar: 10 µm.

Actin cytoskeleton self-organization in the keratinocyte cell line HaCaT, a spontaneously immortalized human cell line from skin that is capable of normal terminal differentiation (Boukamp et al., 1988), was examined. On small adhesive islands, no difference between keratinocytes and fibroblasts was detected since the actin cytoskeleton in both cell types was predominantly circular. As the size of adhesive islands was increased, so too did the proportion of keratinocytes able to develop RFs in the radial pattern (Fig. 4A,B).

The total focal adhesion area in HaCaT cells was drastically smaller than in fibroblasts (Fig. 4C,D compared to Fig. 1D,E). Analysis of variance (one-way ANOVA with Tukey's multiple comparison test) revealed significant differences between mean total focal adhesion area of fibroblasts and keratinocytes on all pattern sizes (*P*<0.0001), except for the smallest pattern area (500 µm^2^) where there was no significant difference. However, similar to fibroblasts, keratinocytes confined to circular fibronectin islands also demonstrated dependence of total focal adhesion area on the projected area of the cell. There is a statistically significant difference between the total focal adhesion area in two groups of sizes: small (500 and 750 µm^2^) and relatively large (1000, 1500, 2000 and 2500 µm^2^) adhesive islands (Fig. 4C).

**Fig. 5.**
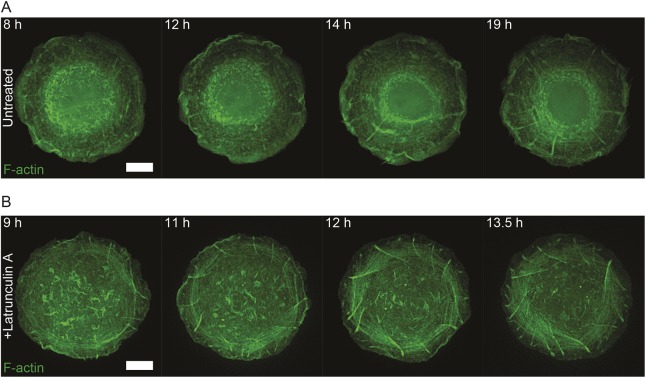
**Induction of chiral actin swirling in keratinocytes treated with low doses of latrunculin A.** (A) Representative time-lapse series showing actin cytoskeleton development in HaCaT cells expressing LifeAct–GFP seeded on 1800 µm^2^ fibronectin-coated islands and imaged overnight. Time in hours after seeding is indicated in the upper left corner of frames. See Movie 5. Scale bar: 10 µm. (B) Representative time-lapse series showing development of the chiral actin pattern in HaCaT cells expressing mRuby–LifeAct (pseudo-colored green) that were treated with 20 nM latrunculin A 8 h after seeding on 1800 µm^2^ fibronectin-coated islands. Time in hours after seeding is indicated in the upper left corner of frames. See Movie 6. Scale bar: 10 µm.

The actin cytoskeleton self-organization in keratinocytes only develops as far as the radial pattern and never showed transitions to the chiral actin pattern (Figs 4B, 5A; Movie 5). The linear actin pattern (final stage of actin cytoskeleton development in fibroblasts) was never observed in HaCaT cells plated and imaged overnight on fibronectin islands. Similar to the situation in EGF-treated NBT-II cells, EMT induction in HaCaT cells was insufficient to induce chirality in the actin cytoskeleton (Fig. S3D–F).

HaCaT cells, however, have the potential to develop a chiral organization of the actin cytoskeleton. We found that treatment of these cells with a very small dose of the actin-monomer-binding drug latrunculin A was sufficient to trigger the development of the chiral actin pattern (Fig. 5B; Movie 6). In latrunculin A-treated keratinocytes filmed overnight on 1800 µm^2^ fibronectin islands, 12 out of 40 cells demonstrated chiral actin cytoskeleton swirling. Unexpectedly, the directionality of chiral swirling induced by latrunculin A treatment was clockwise (Fig. 5B; Movie 6), the opposite to the ‘normal’ counter-clockwise swirling that spontaneously emerges in HFFs (Movie 1 and Tee et al., 2015).

### Molecular requirements for development of the chiral actin pattern in keratinocytes: functions of DIAPH1 formin and VASP

In search of the molecular players participating in the latrunculin A-induced development of chiral actin patterns in keratinocytes, we assessed how other drugs as well as some genetic manipulations affect this process. First, we demonstrated that latrunculin A-mediated induction of chiral actin pattern development does not depend on any transcription-mediated effects. Indeed chiral actin patterns developed with the same efficiency in cells pre-treated with actinomycin D for 2 h and incubated with actinomycin D during latrunculin A treatment as in control cells treated with latrunculin A only (Fig. S5A,B). The concentration of actinomycin D used (2 µg ml^−1^) was sufficient to block the incorporation of uridine into the macromolecules of treated cells (Fig. S5C,D).

We assessed the effect of two inhibitors of actin polymerization, cytochalasin D and SMIFH2 on formation of radial fibers and development of the chiral actin pattern in keratinocytes. Cytochalasin D, an inhibitor of actin filament elongation, did not affect actin pattern formation at the tested low concentrations selected to avoid bulk changes in actin cytoskeleton organization (Fig. S6A). These concentrations, however, produced an effect on cell mechanics similar to that of latrunculin B in another cellular system (Wakatsuki et al., 2001). At the same time, the specific inhibitor of formin-dependent actin filament nucleation SMIFH2, interfered significantly with formation of radial fibers in keratinocytes (Fig. S6A), which is consistent with the similar effect on fibroblasts described previously (Tee et al., 2015). Moreover, SMIFH2 prevented the shift to chiral actin patterns induced in keratinocytes by latrunculin A (Fig. S6A).

**Fig. 6.**
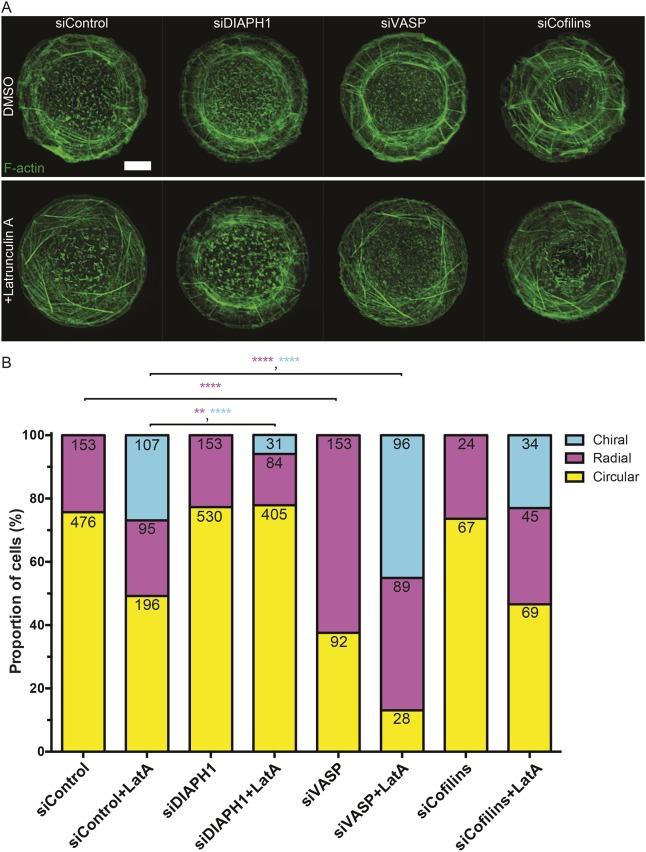
**Chiral actin pattern development in latrunculin A-treated keratinocytes is regulated by DIAPH1 and VASP.** (A) Images showing actin cytoskeleton organization (phalloidin stained) in HaCaT cells genetically silenced with scramble (Control) siRNAs or siRNAs targeting DIAPH1, VASP and cofilins (1 and 2 together). siRNA-transfected cells were treated with DMSO (upper panel) or 100 nM Latrunculin A (LatA, lower panel) and fixed after overnight seeding on 1800 µm^2^ fibronectin-coated islands. Scale bar: 10 µm. (B) Bar chart showing fractions of cells with actin cytoskeleton demonstrating circular, radial or chiral pattern from cells imaged under conditions of A. Numbers of cells displaying each actin pattern are labeled on top of each bar per condition, pooled from three to six independent experiments. Fisher's exact tests were used to assess significance between the fractions of cells displaying radial (magenta asterisk) or chiral (cyan asterisk) actin pattern in pairs (siControl vs siDIAPH1, siControl vs siVASP, siControl vs siCofilins, siControl+LatA vs siDIAPH1+LatA, siControl+LatA vs siVASP+LatA, siControl+LatA vs siCofilins+LatA); **P<0.01, ****P<0.0001.

We further examined the effects of depletion of several key regulators of actin polymerization and depolymerization on self-organization of the actin cytoskeleton in HaCaT keratinocytes plated on 1800 µm^2^ circular adhesive islands (Fig. 6A; Fig. S6B). We have found that siRNA-mediated knockdown of the major formin family protein DIAPH1 did not prevent formation of radial actin fibers but significantly decreased the induction of chiral actin pattern development by latrunculin A (Fig. 6B). The depletion of another stimulator of actin filament polymerization, VASP, surprisingly produced the opposite effect (Fig. 6B). VASP knockdown significantly augmented the fraction of radial cells in untreated keratinocytes, as well as the fraction of chiral cells in keratinocytes treated with latrunculin A. The depletion of cofilins (1 and 2 together) did not significantly affect the fractions of either radial cells or chiral cells induced by latrunculin A (Fig. 6B). Thus, DIAPH1-driven actin polymerization is indispensable for the development of chiral actin patterns, while VASP is a potent regulator of both radial fiber formation and chiral actin pattern development upon latrunculin A treatment.

### The nucleus and keratin filament network follow chiral swirling of the actin cytoskeleton

The nucleus is associated with the actin cytoskeleton by a number of molecular links (Kim et al., 2014; Lele et al., 2018; Maninová and Vomastek, 2016) and one possible cause of latrunculin A-mediated induction of chiral actin swirling is that latrunculin A treatment weakens such links thereby facilitating the actin cytoskeleton rotational movement. Here, we have shown, however, both in fibroblasts demonstrating spontaneous actin swirling and in keratinocytes demonstrating swirling triggered by latrunculin A the nuclei preserved their interactions with the actin cytoskeleton during swirling and rotated together with the actin cytoskeleton.

**Fig. 7.**
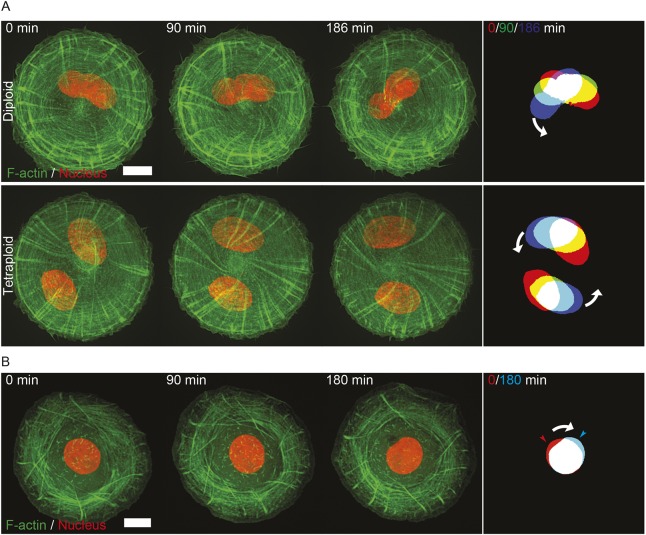
**Nuclear rotation follows the direction of chiral actin swirling in fibroblasts and latrunculin A-treated keratinocytes.** (A) Representative time-lapse series showing actin cytoskeleton development and nucleus rotation in diploid (upper panel, untreated cells) or tetraploid (lower panel, achieved by pre-treatment with cytochalasin D, see Materials and Methods) HFF cells expressing LifeAct–GFP with the nuclear marker (BFP-NLS) seeded on 1800 µm^2^ fibronectin-coated islands. Time in minutes after start of imaging is indicated in upper left corner of each frame. See Movie 7. The final frame on each panel shows a color-coded overlay of threshold images of nuclei at 90 min intervals with white arrows to show the direction of nuclei movement. Scale bar: 10 µm. (B) Representative time-lapse series showing actin cytoskeleton development and nucleus rotation in latrunculin A-treated HaCaT cells expressing LifeAct–GFP with the histone marker (H1–RFP) for nucleus visualization, seeded on 2000 µm^2^ fibronectin-coated islands. 200 nM of latrunculin A was added to cells 40 min after seeding. Time in minutes after start of imaging is indicated in upper left corner of each frame. See Movie 8. The final frame shows a color-coded overlay of threshold images of nuclei at the first (red) and last (cyan) frame with arrowheads marking the tip of the nucleus. The white arrow shows the direction of nucleus movement. Scale bar: 10 µm.

The chiral swirling in fibroblasts was accompanied by rotation of their nuclei as could be seen through BFP-NLS labeling (Fig. 7A, upper panel; Movie 7). Nuclei are rotating counter-clockwise following the cytoskeleton swirling direction. Removal of the nuclei by treatment with 8 µg ml^−1^ cyotchalasin B followed by high-speed centrifugation and recovery in normal medium, did not interfere with spontaneous chiral swirling of the actin cytoskeleton in fibroblasts (Fig. S7A), strongly suggesting that nuclear rotation is a consequence of the actin swirling and not vice versa. Of note, enucleation also did not apparently increase the rate of cytoskeleton swirling or fraction of the swirling cells (Fig. S7B). Moreover, artificial increase of the number of nuclei by long-term pre-treatment of cells with cytochalasin D to prevent cytokinesis did not apparently slow down chiral swirling (Fig. 7A, lower panel). Thus, the association between the actin cytoskeleton and the nucleus is preserved during actin cytoskeleton rotation and does not interfere with development of the swirling actin pattern in fibroblasts. In keratinocytes treated with small doses of latrunculin A, clockwise chiral swirling of the actin cytoskeleton is similarly accompanied by clockwise rotation of the nucleus (Fig. 7B; Movie 8), suggesting that in this cellular system the coupling between the actin cytoskeleton and the nucleus is also preserved during actin swirling and does not prevent it.

**Fig. 8.**
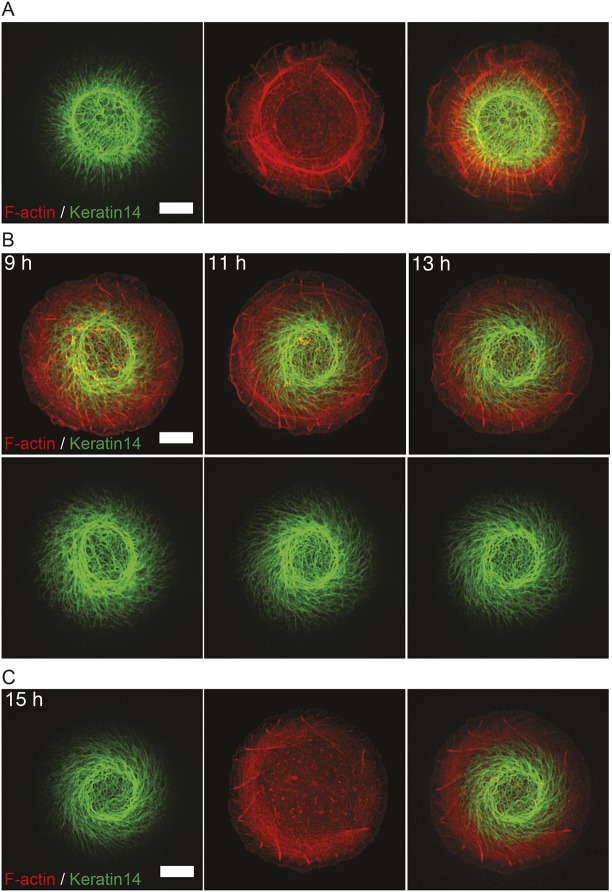
**Interplay between cytokeratin and actin cytoskeleton self-organization in keratinocytes confined to adhesive islands.** (A) Representative frame from a time-lapse series showing cytoskeleton development in HaCaT cells expressing mEmerald–Keratin14 with mRuby–Lifeact seeded on 1800 µm^2^ fibronectin-coated islands. See Movie 9. Scale bar: 10 µm. (B) Representative time-lapse series showing cytoskeleton progression in latrunculin A-treated HaCaT cells expressing mEmerald–Keratin14 with mRuby–Lifeact seeded on 1800 µm^2^ fibronectin-coated islands. 20 nM of latrunculin A was added to cells 8 h after seeding. Time in hours after seeding is indicated in upper left corner of each frame. See Movie 10. Scale bar: 10 µm. (C) Representative frame from time-lapse series shown in B taken at 15 h after seeding. See Movie 10. Scale bar: 10 µm.

The keratin filament network is the most abundant and diverse cytoskeletal system in epithelial cells (Windoffer et al., 2011), and during the process of EMT endogenous expression of keratin is downregulated in favor of vimentin expression (Boyer et al., 2000; Nieto et al., 2016; Savagner, 2010; Tan et al., 2014). Previous studies have revealed the molecular links between the keratin filament network and both the nucleus and the actin cytoskeleton (Pan et al., 2013). In keratinocytes plated on 1800 µm^2^ fibronectin-coated islands, the assembly of keratin filaments follows the spatiotemporal pattern previously described (Kölsch et al., 2010). Filaments begin to form near the cell periphery and then move centripetally to be incorporated into a dense network in the central part of the cell (Fig. 8A; Movie 9). The chiral swirling of the actin cytoskeleton in keratinocytes induced by mild latrunculin A treatment (Fig. 5B) made it possible to investigate the relationship between keratin and actin filament swirling. Our observations showed that in keratinocytes demonstrating chiral actin swirling, the keratin network followed the swirling movement of actin (Fig. 8B; Movie 10) and organized itself into a chiral pattern (Fig. 8C).

Despite the clear interplay between the actin and keratin cytoskeletons, the removal of the keratin network did not obviously affect actin cytoskeleton self-organization. We compared actin cytoskeleton dynamics in a normal murine keratinocyte cell line and in a line lacking all keratins (Seltmann et al., 2013; Vijayaraj et al., 2009). Immunofluorescence staining revealed that normal murine keratinocytes indeed contained filament networks that were positive for keratin 5, while knockout keratinocytes were negative for keratin 5 staining (Fig. S8A). The actin cytoskeleton patterns visualized in both the murine keratinocyte lines 1 day after plating on collagen-coated islands were essentially similar to the actin cytoskeleton self-organization in the human keratinocyte cell line HaCaT on fibronectin-coated islands (Fig. S8B compared to Fig. 4A). In both wild-type and knockout murine keratinocytes plated on small collagen islands (500, 750 and 1000 µm^2^) the circular actin pattern dominated. In both wild-type and knockout keratinocytes plated on large collagen-coated islands (1500, 2000 and 2500 µm^2^), the radial actin pattern developed in the majority of cells (Fig. S8B), with a slightly higher rate in knockout cells as compared to wild-type murine keratinocytes (Fig. S8C). However, we did not observe the development of the chiral actin pattern in either wild-type or keratin-knockout murine keratinocytes (Fig. S8B,C).

## DISCUSSION

Our study has revealed clear and reproducible differences between the development of the actin cytoskeleton in fibroblasts and epithelial cells. Such differences were evident in a very simple experimental system when cells are deprived of contacts with each other and of the possibility to migrate. This approach helped us to dissect the intrinsic potential of the actin cytoskeleton for self-organization from the effects that emerge due to cell shape changes and cell migration. Recent studies of actin cytoskeleton self-organization in fibroblasts confined to circular adhesive islands revealed a rich repertoire of cytoskeletal patterns that can be generated as well as rules underlying the development of these patterns in time (Tee et al., 2015). In the present study, we have analyzed the actin cytoskeleton self-organization in a variety of epithelial cell lines under conditions of confinement to circular adhesive islands.

Among the basic parameters that could affect the actin cytoskeleton self-organization is the cell spreading area. Upon spreading under unconstrained conditions the maximal cell spreading area should scale with parameters related to cell size, such as cell volume and surface area. Indeed multinucleated cells plated on planar substrates always have larger projected cell areas than their mononucleated counterparts (Lyass et al., 1984). The interrelationship between the projected cell area and organization of the actin cytoskeleton, development of traction forces and assembly of focal adhesions was observed in many prior studies (Han et al., 2012; Oakes et al., 2014; Rape et al., 2011; Wang et al., 2016). Therefore, the optimal development of the actin cytoskeleton requires a sufficient spreading area that could be different for different cell types. In order to properly compare the morphogenetic potential of the actin cytoskeleton in different cell types, it is essential to investigate the process in cells confined to adhesive islands with different areas.

We found that all cells confined to adhesive islands with a low projected cell area organized their actin cytoskeleton into a circular actin pattern regardless of type. Differences in actin cytoskeleton development only became apparent in cells confined to large adhesive islands when three distinct types of behavior were identified in fibroblasts, keratinocyte epithelial cells and epithelial cells not originating from skin. When plated on sufficiently large adhesive islands, fibroblasts are able to efficiently reproduce the full set of actin patterns described previously by progressing to the symmetric radial and then breaking symmetry into the chiral and linear actin patterns (Tee et al., 2015). In contrast, keratinocytes are only able to develop as far as the radial actin pattern, while non-keratinocyte epithelial cells cannot even develop past the circular pattern. EMT rescued the inability of non-keratinocyte epithelial cells to transition to a radial actin pattern by promoting the growth of radial fibers, but remained insufficient to induce development of left–right asymmetry typical of fibroblasts under these conditions.

Downregulation of cytokeratins and disappearance of the keratin filament network are hallmarks of EMT in many epithelial cell types (Savagner, 2010). Indeed, keratinocytes differ from fibroblasts because they express significant amounts of keratin intermediate filament proteins that are organized into well-developed filament networks (Purkis et al., 1990). Keratin filaments are physically connected to the actomyosin network (Kölsch et al., 2009; Wöll et al., 2005). Nevertheless, we have found that depletion of keratin proteins (Seltmann et al., 2013), which resulted in the disappearance of the keratin filament network, did not change the actin filament organization in murine keratinocytes. It still remains uncertain whether deficiencies in the development of the actin cytoskeleton of epithelial cells depend instead on the absence of another intermediate filament protein, namely vimentin. Of note, vimentin filaments have also been shown to follow actin reorganizations rather than determine them (Dupin et al., 2011; Jiu et al., 2015), similar to what was seen for the keratin filaments that we characterized here.

The main factors participating in self-organization of the actin cytoskeleton are nucleation of polymerization of actin filaments forming radial actin bundles and centripetal flow caused by myosin II-driven contractility of transverse circumferential actin bundles. Focal adhesions are the cytoskeletal domains that efficiently nucleate the assembly of actin filaments. At the early stages of focal adhesion assembly they are associated with proteins of the Arp2/3 complex (Beckham et al., 2014; Chorev et al., 2014; DeMali et al., 2002), while mature focal adhesions nucleate the associated actin bundles via formin activity (Hotulainen and Lappalainen, 2006; Oakes et al., 2012; Riveline et al., 2001; Tee et al., 2015). The focal adhesions may not only generate radial fibers but also induce their tilting, which finally leads to the development of chiral swirling. The formins localized to focal adhesions could be responsible for filament twisting coupled with polymerization, which can underlie the mechanism of radial fiber tilting (Tee et al., 2015).

Since focal adhesions are mechanosensitive structures (Balaban et al., 2001), the larger focal adhesion size in cells with larger projected cell areas could be a consequence of stronger traction forces that the radial fibers in these cells exert on focal adhesions (Rape et al., 2011; Reinhart-King et al., 2005). This effect can be explained based on observations that longer radial fibers interact with a larger number of contractile myosin IIA-containing transverse fibers (Tee et al., 2015) and therefore may transmit stronger forces to the focal adhesions. Keratinocytes form shorter radial fibers and, probably because of that, have smaller focal adhesions than fibroblasts, which could be responsible for the lack of transition from the radial to the chiral actin pattern in keratinocytes.

Keratinocytes do have the potential to generate chiral swirling as can be seen in experiments with induction of swirling by low doses of latrunculin A. Even though the direction of latrunculin A-induced swirling in keratinocytes is opposite to that of fibroblasts, the resultant actin pattern is qualitatively similar to that of fibroblasts. Latrunculin A is a drug that specifically affects actin polymerization by sequestering G-actin monomers (Ayscough, 1998; Ayscough et al., 1997; Coué et al., 1987; Morton et al., 2000), but since actin monomers can affect transcription via myocardin-related transcription factor, MRTF (Sotiropoulos et al., 1999; Treisman, 2013), some latrunculin A effects could be in principle mediated by transcriptional regulation. We have shown, however, that latrunculin A can induce actin chiral swirling in keratinocytes under conditions of transcriptional repression mediated by actinomycin D. This suggests that the chirality induction is instead a direct effect of the drug on the actin cytoskeleton structure and dynamics.

Since cofilins are known mediators of actin depolymerization, we checked whether depletion of these proteins would affect chirality induction in the presence or absence of latrunculin A. However, we did not find any apparent changes in organization of the actin cytoskeleton after double knockdown of cofilin 1 and 2. It is possible that this lack of a noticeable phenotype is due to a redundancy between cofilins and actin depolymerizing factor (ADF) as was previously documented (Hotulainen et al., 2005).

The small doses of latrunculin A used in the present study did not apparently produce a disruptive effect in the bulk actin cytoskeleton. One can suggest, however, that latrunculin A disrupts some links between the actin cytoskeleton and certain components of the cell, which may normally oppose actin swirling. Our experiments revealed surprisingly that two possible candidates for the role of such components (the nucleus and the cytokeratin network) in fact rotate themselves following the direction of actin swirling. Moreover, neither the removal nor artificial increase of the number of nuclei in fibroblasts affected the transition from radial to swirling actin organization. Similarly, the knockout of cytokeratins in keratinocytes did not induce spontaneous development of actin swirling. Thus, if even a hypothesis on weakening the links between potentially rotatable and immobile intracellular domains by latrunculin A is correct, neither the nucleus nor the cytokeratin network function as these immobile structures.

We have demonstrated that the transition from the radial to the chiral actin organization triggered by latrunculin A in keratinocytes did not occur in cells lacking the formin DIAPH1, but was strongly facilitated in cells lacking VASP protein. These observations strongly suggest that the phenomenon of chiral swirling depends on regulation of actin polymerization and attracts attention to collaboration/competition between VASP and formin proteins. The involvement of formins in establishment of actin cytoskeleton chirality was proposed in our previous publication (Tee et al., 2015), and is supported by more recent findings showing that a Diaphanous family formin is responsible for establishment of left–right asymmetry in snails (Davison et al., 2016; Kuroda et al., 2016). It was also noticed that small doses of latrunculin B (having apparently the same mechanism of action as latrunculin A; Wakatsuki et al., 2001) can potentiate the activity of formin via an increase in free G-actin, which stimulates formin activity (Higashida et al., 2008). Thus, latrunculin A-mediated induction of actin chiral swirling in our experiments could be a consequence of activation of DIAPH1. Interestingly, the activity of Diaphanous formins is also indispensable for EMT in several types of epithelial cells (Rana et al., 2018).

Diaphanous formins can cooperate with VASP in regulation of actin polymerization and serum response factor activity; moreover, these proteins can physically interact with each other (Grosse et al., 2003). In other systems, such as *Drosophila*, interactions between Diaphanous formins and Ena/VASP could be antagonistic (Bilancia et al., 2014; Nowotarski et al., 2014). Thus, facilitation of latrunculin A-induced transition of actin cytoskeleton organization in keratinocytes by VASP knockdown can be mediated by a net increase in DIAPH1 activity in these cells. Of note, knockdown of VASP promoted the formation of radial fibers (a prerequisite to the transition to chiral actin swirling) in untreated keratinocytes. Clarification of the details of these mechanisms will require further investigation.

Even though our study shows that development of left–right actin asymmetry *in vitro* is less typical in epithelial cells than in fibroblasts, we have demonstrated that it is in principle possible to induce such asymmetry. Of note, the development of left–right asymmetry in embryogenesis *in vivo* often depends on epithelial cell asymmetry. In *Drosophila*, asymmetric tissue rotation during development of the hindgut (Hatori et al., 2014; Taniguchi et al., 2011) or male genitalia (Sato et al., 2015) depends on the intrinsic asymmetry of epithelial cells. Moreover, global asymmetry in positioning of visceral organs in vertebrates depends on development of left–right asymmetric polarity in a group of epithelial cells in the left–right organizer, known as the node, the Hensen's node or the Kupffer's vesicle in different species (Blum and Ott, 2018). It is becoming increasingly clear that the molecular motor myosin 1D is a conserved determinant in the development of left–right asymmetry in many systems (Hozumi et al., 2006; Juan et al., 2018; Saydmohammed et al., 2018; Spéder et al., 2006; Tingler et al., 2018). *In vitro*, myosin 1D generates asymmetric counter-clockwise gliding of actin filaments (Lebreton et al., 2018), but the cellular mechanism behind emerging asymmetry in the actin cytoskeleton remains obscure. Elucidation of the role of formin-dependent actin polymerization in establishment of cell left–right asymmetry is an inspiring avenue for future studies.

## MATERIALS AND METHODS

### Cell culture

All cells were maintained in a humidified incubator set at 37°C and atmospheric 5% CO_2_. Human foreskin fibroblasts (HFFs) were cultured and transfected as previously described (Tee et al., 2015). Source of ovarian cancer cell lines (PEO1, OVCA429, SKOV3 and HEYA8) can be referred to in Huang et al. (2013), while HaCaT and MDCK are in Vedula et al. (2014), and NBT-II in Chua et al. (2012). MCF-7 and MCF-10A were gifts from the B.C. Low Group, Mechanobiology Institute, Singapore while Caco-2 was gifted by the R. Zaidel-Bar Group, Mechanobiology Institute, Singapore. NBT-II, MCF-7, HaCaT, Caco-2, MDCK, SKOV3 and OVCA429 cells were cultured in high-glucose Dulbecco's modified Eagle's medium (DMEM, Invitrogen) supplemented with 10% heat inactivated fetal bovine serum (HI-FBS, Invitrogen), 1 mM sodium pyruvate (Invitrogen) and 0.1% penicillin and streptomycin (P/S, Invitrogen). MCF-10A cells were cultured in DMEM with nutrient mixture F12 (DMEM/F12, Invitrogen) supplemented with 5% horse serum (Invitrogen), 20 ng ml^−1^ epidermal growth factor (EGF, Peprotech), 0.5 µg ml^−1^ hydrocortisone (Sigma), 100 ng ml^−1^ cholera toxin (Sigma), 10 mg ml^−1^ insulin (Sigma) and 0.1% P/S. Murine keratinocytes (wild type and knockout, kind gifts from Thomas M. Magin, University of Leipzig, Germany) were cultured in low Ca^2+^ DMEM/Ham's F12 (Merck) supplemented with 10% chelex-treated HI-FBS, 10 ng ml^−1^ EGF, 0.5 µg ml^−1^ hydrocortisone, 10^−10^ M cholera toxin, 2.5 µg ml^−1^ insulin, 0.18 mM adenine (Sigma), 1 mM Glutamax (Invitrogen), 1 mM sodium pyruvate and 0.1% P/S. HEYA8 and PEO1 cells were cultured in Roswell Park Memorial Institute-1640 medium supplemented with 10% HI-FBS and 0.1% P/S.

### Introduction of plasmid and siRNA into cells

Transient transfection of DNA plasmids into cells was achieved by electroporation (Neon transfection system, Life Technologies) following the manufacturer's instructions. Electroporation conditions for NBT-II, MCF-10A and HaCaT were two pulses of 1200 V for 20 ms, while MCF-7 required two pulses of 1250 V. Plasmids encoding the following fluorescence fusion proteins were used: LifeAct–GFP (R. Wedlich-Soldner Group, Max Planck Institute of Biochemistry, Martinsried, Germany), F-tractin–RFP (R. Zaidel-Bar Group, Mechanobiology Institute, Singapore), H1–RFP (G. V. Shivashankar Group, Mechanobiology Institute, Singapore), mRuby-Lifeact and mEmerald-Keratin14 (Michael W. Davidson Group, Florida State University, Tallahassee, FL, USA) as well as Blue Fluorescent Protein-tagged nuclear localization signal (BFP-NLS). For genetic silencing studies, cells were seeded in 35 mm dishes on day 0. On days 1 and 2, cells were transfected with 20 µM of the appropriate small interfering RNA, siRNA: control siRNA (ON-TARGETplus Non-targeting Control Pool siRNA, D-001810-10, Dharmacon), DIAPH1 siRNA (target sequence 5′-GAAGUGAACUGAUGCGUUU-3′, Sigma), VASP pool siRNAs (sc-29516, Santa Cruz Technology, SCT), cofilin 1 pool siRNAs (sc-34078, SCT) and cofilin 2 pool siRNAs (sc-37027, SCT) using Lipofectamine RNAiMAX (Invitrogen) following the manufacturer's instructions. On day 3, cells were trypsinized and left to recover in suspension at a cell density of 6×10^4^ cells ml^−1^ for 1 h in a humidified incubator at 37°C, before seeding onto patterned dishes at a density of 6×10^4^ cells ml^−1^, and the remaining cells were used for immunoblotting.

### Growth factor and drug treatments of epithelial cells

To induce EMT in epithelial cells, NBT-II cells were treated in culture medium supplemented with 100 ng ml^−1^ EGF. For EMT induction in keratinocytes, HaCaT cells were treated in culture medium supplemented with 20 ng ml^−1^ TGF-β1 (Sigma) and 100 ng ml^−1^ EGF for 1 day and refreshed with 10 ng ml^−1^ TGF-β1 and 100 ng ml^−1^ EGF daily for 3 more days before overnight imaging, or for 4 more days before lysate preparation. To induce chirality in keratinocytes, HaCaT cells were treated with 20–200 nM of latrunculin A (Sigma). For drug inhibition studies, 5–20 nM cytochalasin D (Sigma) or 15 µM SMIFH2 (Sigma) were added to HaCaT cells within 2 h of seeding on micropatterned dishes. For transcriptional inhibition study, cells were treated with 2 µg ml^−1^ of actinomycin D (Sigma) or pre-treated with 2 µg ml^−1^ of actinomycin D for 2 h prior to the addition of latrunculin A. All inhibitors remained in the medium during the entire period of observation.

### Generation of enucleated and multinucleated cells

HFFs were enucleated as previously described (Goldman et al., 1973). Briefly, cells or GFP–LifeAct and BFP-NLS double-transfected cells were seeded onto plastic coverslips (ibidi) overnight. Next, cells were treated with 5 ml of 10 µg ml^−1^ cytochalasin B (Sigma) in a 50 ml falcon tube and centrifuged at 10,864 ***g*** (Beckman centrifuge X30R) for 1 h at 37°C to enucleate cells. Cells were washed three times with complete medium and allowed to recover for at least 2 h in complete medium following which, cells were trypsinized for seeding onto the micropatterned substrate for the experiment. Nuclei were either visualized by BFP-NLS transfection or labeled with Hoechst 33342 (10 µg ml^−1^ for 10 min) for live imaging of the nucleus. For selection of cells for live imaging, only cells already demonstrating the radial actin pattern were analyzed. To generate multinucleated cells, HFFs were treated with 1 μM of cytochalasin D for 48 h to block cytoplasmic cleavage during cell division.

### Protein micropatterning of substrates

Cells were seeded on substrates containing circular adhesive islands of various areas (500, 750, 1000, 1500, 2000 and 2500 µm^2^), or circular islands with fixed areas (700, 1200 or 1800 µm^2^). Adhesive circular islands were fabricated by using a PDMS stamp through either micro-contact printing as described previously (Tee et al., 2015), or, by a slightly modified version of stencil patterning (Masters et al., 2012). For stencil patterning, PDMS stamps were first inverted and placed onto a hydrophobic uncoated 35 mm µ-dish (ibidi). Norland Optical Adhesive 73 (NOA-73, Norland Inc.) was deposited along an edge of the stamp and allowed to fill in the gaps between the PDMS stamp and dish by capillary action. The NOA stencil was cured under ultraviolet illumination for ∼15 s. After peeling off the PDMS stamp, the stencil and dish were incubated with fibronectin (Calbiochem, Merck Millipore) or collagen I (BD Biosciences) at a concentration of 50 µg ml^−1^ in PBS or acetic acid, respectively, at 4°C overnight. Unadsorbed protein was rinsed off, the NOA stencil removed and the dish was then passivated with 0.2% pluronic acid in water for 10 min at 37°C. Finally, dishes were rinsed in PBS three times before epithelial cells were seeded at a density of 6×10^4^ or 7×10^4^ cells ml^−1^, while fibroblasts were seeded at 5×10^4^ cells ml^−1^.

### Immunofluoroscence

Cells were fixed with 4% paraformaldehyde in PBS for 10 min, or by 100% methanol for 5 min, followed by three PBS washes. Cells fixed with paraformaldehyde were permeabilized with 0.5% Triton X-100 and subsequently quenched with 0.1 M glycine in PBS for 10 min each. After PBS washes, blocking was performed with 2% BSA in PBS for 1 h at room temperature (RT) prior to overnight primary antibody incubation at 4°C with mouse anti-paxillin (Cat. no. 610569, 1:100, BD Biosciences) or anti-β-actin (AC-15, 1:200, Sigma) in 2% BSA in PBS. Fixed cells were washed with PBS three times and then incubated with an appropriate Alexa Fluor-conjugated mouse secondary antibody (1:250 dilution, Thermo Fisher Scientific) in 2% BSA in PBS for 1 h at RT. F-actin staining was performed using Alexa Fluor 488 (Thermo Fisher Scientific)- or TRITC (Sigma)-conjugated phalloidin at a dilution of 1:500 while Keratin5 staining was performed using anti-cytokeratin 5 conjugated to Alexa Fluor 647 at a dilution of 1:100 (EP1601Y, Abcam), incubated overnight at 4°C or 1 h at RT. Incorporation of 5-ethynyl uridine into newly synthesized RNA was visualized by following the manufacturer's protocol with a Click-iT^®^ RNA Imaging Kit (C10330, Thermo Fisher Scientific).

### Immunoblotting

Cells were lysed with RIPA buffer (Sigma) and extracted proteins were separated by 4–20% gradient SDS-polyacrylamide gel electrophoresis (Thermo Fisher Scientific) and transferred to a 0.2 µm PVDF membrane (Bio-Rad) at 100 V for 1.5 h. Membranes were blocked with 5% non-fat milk (Bio-Rad) in Tris-buffered saline with 0.1% Tween 20 (TBST) for 1 h at RT before incubation with the appropriate primary antibody [mouse anti-GAPDH (6C5, SCT) at a dilution of 1:3000, mouse anti-E-cadherin (Cat. no. 610181, BD Transduction) at a dilution of 1:5000, rabbit anti-slug (C19G7, Cell Signaling Technology, CST) at a dilution of 1:250, rabbit anti-vimentin (D21H3, CST) at a dilution of 1:250, mouse anti-VASP (A-11, SCT) at a dilution of 1:1000, mouse anti-cofilins (E-8, SCT) at a dilution of 1:1000, mouse anti-Dia1 (Cat. no. 610848, BD Biosciences) at a dilution of 1:1000] in 5% non-fat milk in TBST at 4°C overnight. After washes in TBST, the membrane was incubated with an appropriate HRP-conjugated secondary antibody (Bio-Rad) at a dilution of 1:2000 in 2.5% non-fat milk in TBST. The membrane was then processed for ECL detection (Bio-Rad) and chemiluminescence was detected in the Bio-Rad ChemiDoc Imaging System.

### Imaging, processing, characterization and statistical analysis

For live imaging, cells cultured in Phenol Red-containing DMEM were switched to Leibovitz's L-15 (supplemented with 10% FBS and 1 mM sodium pyruvate), while MCF-10A cells were imaged in normal culture medium buffered by supplementing a final concentration of 20 mM HEPES (Invitrogen) and 0.369% sodium bicarbonate (Invitrogen). Time-lapse imaging was performed at imaging rates of 2–10 min frame^−1^ with *z*-stacks (10–15 µm) of step size 0.35 µm, on a spinning-disc confocal microscope (PerkinElmer Ultraview VoX) on an inverted microscope (Olympus IX81) using 60× (UPlanSApo, NA 1.2, water) or 100× (UPlanSApo, NA 1.4, oil) objectives. Image acquisition was set up in Volocity software and taken by an EMCCD camera (Hamamatsu C9100-13). Brightfield and widefield images where taken on the EVOS FL Imaging system (Thermo Fisher Scientific) using the 4× objective, or, on an Olympus IX81 inverted microscope, with 100 W mercury lamp light source using the 20× (PL-FL, NA 0.45) objective.

Maximum intensity projection through the *z*-stack of time-lapse images were made in Volocity before exporting as TIFF files for further processing in ImageJ (National Institutes of Health, NIH). Image processing functions applied to whole images included: bleach correction, gaussian blur filter, sharpen and subtract background. For measurement of cell spread area and total focal adhesion area, segmentation and particle detection of single-plane phalloidin and paxillin-stained images was performed in ImageJ using the ‘Adaptive 3D Threshold’ and ‘Analyze Particles’ functions, respectively. Segmentation of nuclei was performed in ImageJ using the ‘Maximum Entropy Threshold’ function.

For characterization of cells displaying the circular, radial or chiral actin pattern from live imaging experiments, the most advanced stage of actin pattern development displayed during the time course was selected. Graph plotting and statistical analysis were performed in Prism. Analyses of significant differences between proportions of cells displaying radial or chiral actin patterns were carried out using a Fisher's exact test. Analyses of significant differences between total focal adhesion areas were carried out using a two-tailed Mann–Whitney test or one-way ANOVA with Tukey's multiple comparison test for more than two groups. Statistical methods and sample size were specified in figure legends and differences were accepted as significant if *P*<0.05. No statistical method was used to predetermine sample size.

## Supplementary Material

Supplementary information
