# Supplementary material for: Actin cytoskeleton self-organization in single epithelial cells and fibroblasts under isotropic confinement
Source: J Cell Sci. 2019 Mar 7;132(5):jcs220780. doi: 10.1242/jcs.220780 (PMC6432717; doi:10.1242/jcs.220780)
Supplement: Supplementary information [file joces-132-220780-s1.pdf]

Supplemental Figures

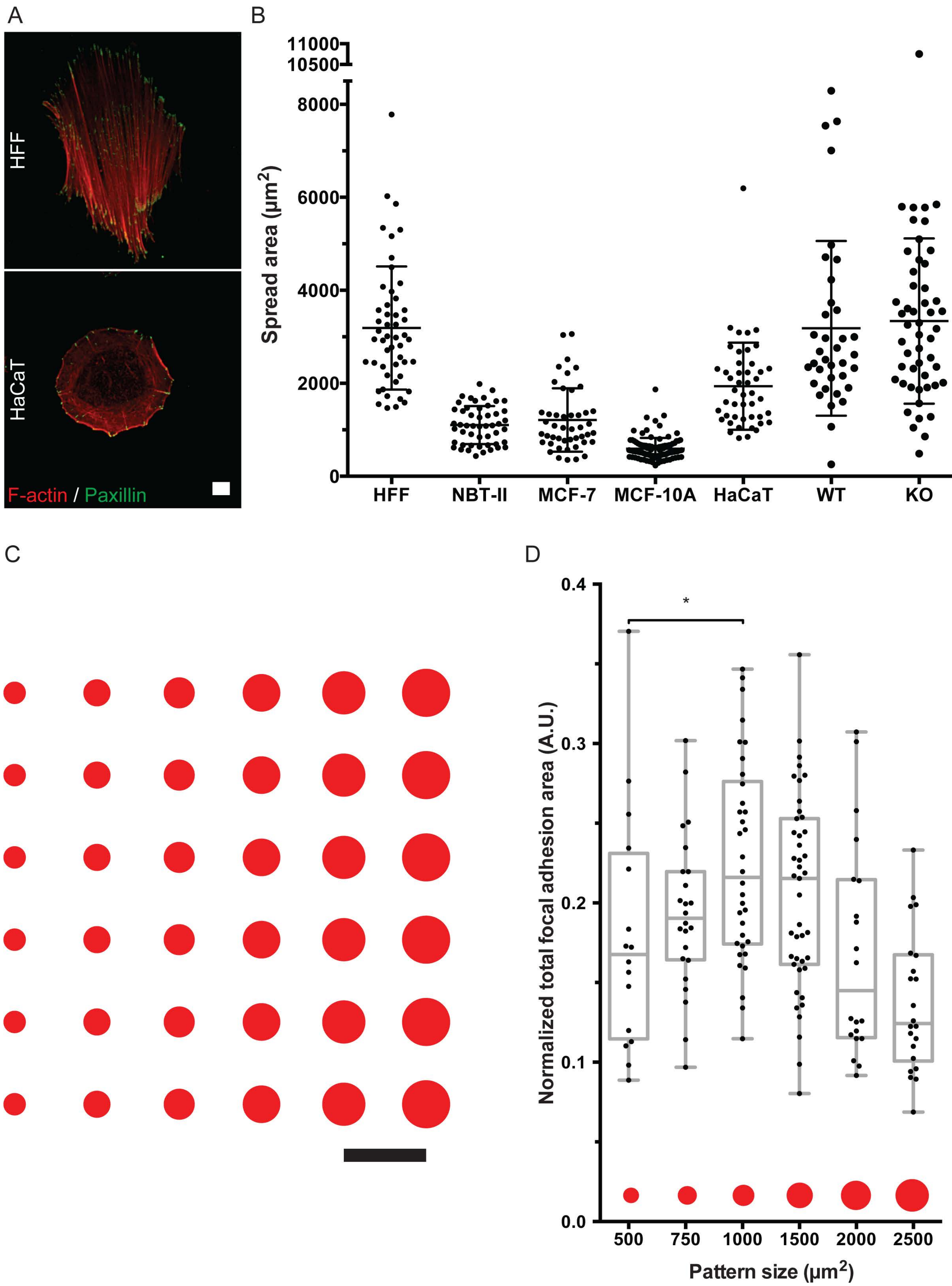

Journal of Cell Science • Supplementary information

Figure S1: Cell morphology and spread area variation between epithelial cell lines and fibroblasts

A) Representative images showing focal adhesion (paxillin immunostaining) and F-actin (phalloidin staining) distribution in HFF and HaCaT fixed 24 h after seeding on planar fibronectin-coated surfaces. Scale bar: 10 μm.

B) Scatter dot plot with Mean $\pm$ Standard Deviation showing cell spread area of different cell lines on planar protein-coated surfaces. HFF, NBT-II and HaCaT were fixed and stained with phalloidin 24 h after seeding on planar fibronectin-coated surfaces. Wild-type (WT) and Knockout (KO) murine keratinocytes were fixed and stained with phalloidin 22 h after seeding on planar collagen-coated surfaces. MCF-7 and MCF-10A were fixed and stained with phalloidin 18 h after seeding on planar collagen-coated surfaces.

C) Schematic showing pattern consisting of six different sizes of circular islands that was used for protein patterning. Scale bar: 50 μm.

D) Box and whiskers plot showing the total focal adhesion area in cells imaged under conditions of Fig. 1E normalized by projected cell area. Mann-Whitney tests were used to assess significance between the normalized total focal adhesion areas in pairs of island sizes; $*P<0.05$. The box represents the 25–75th percentiles, and the median is indicated. The whiskers show the complete range from minimum to maximum values. Points superimposed on the graph show all values.

Journal of Cell Science • Supplementary information

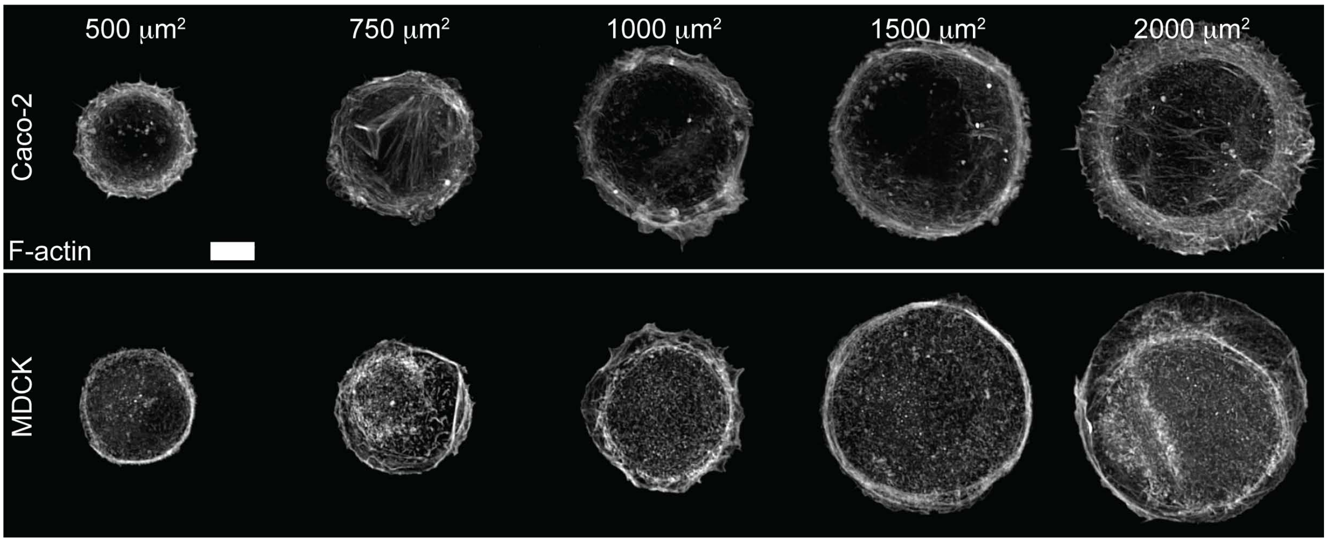

Figure S2: Actin cytoskeleton development in non-keratinocyte epithelial cell lines

Representative images showing actin cytoskeleton organization (phalloidin stained) in Caco-2 and MDCK fixed 20-24 h after seeding on collagen-coated islands of different sizes (500, 750, 1000, 1500, 2000 and 2500 μm^2). Scale bar: 10 μm.

Journal of Cell Science • Supplementary information

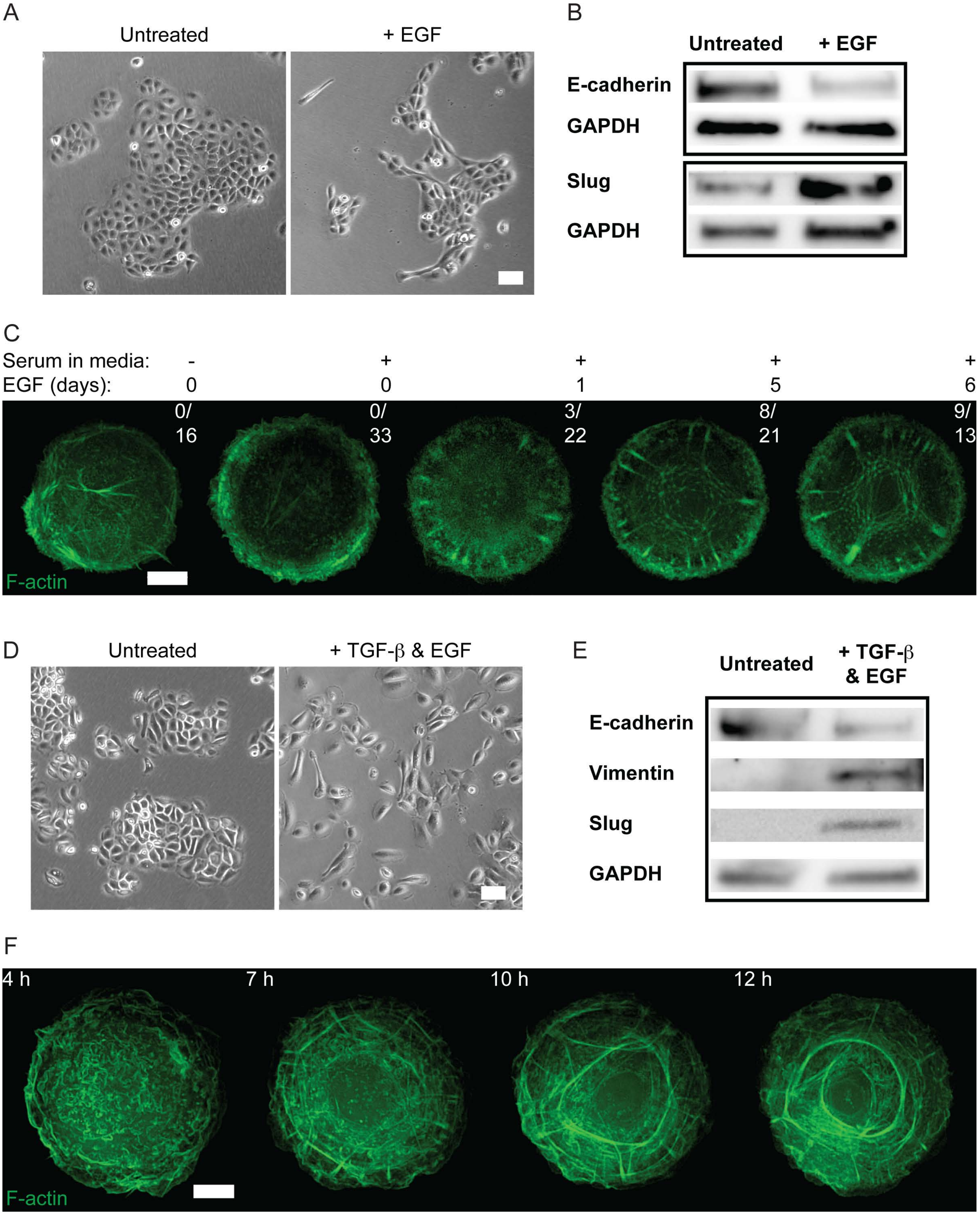

Journal of Cell Science • Supplementary information

Figure S3: Actin cytoskeleton development during epithelial-mesenchymal transition in rat bladder carcinoma cells and human keratinocytes

A) Representative brightfield images of NBT-II in standard culture after cells were left untreated (Untreated, left panel) or treated with 100 ng ml^{-1} EGF for 3 days (+EGF, right panel). Scale bar: 100 μm.

B) Western blot images showing protein expression levels of E-cadherin, Slug and GAPDH in total protein lysates taken from NBT-II cells without (Untreated) or with (+EGF) 100 ng ml^{-1} EGF treatment for 3 days.

C) Representative frames of actin cytoskeleton organization in NBT-II cultured in serum free or serum containing media with or without 100 ng ml^{-1} EGF pre-treatment (in days). Cells were transfected with Lifeact-GFP one day before replating and imaging overnight on 1200 μm^2 fibronectin-coated islands. Fractions of cells able to develop radial fibres (over total number of imaged cells) are shown in the top right corner of each frame representing a treatment condition. Scale bar: 10 μm.

D) Representative brightfield images of HaCaT in standard culture after cells were left untreated (left panel), or, treated with EGF and TGF-β for 3 days (as described in Materials and methods, right panel). Scale bar: 100 μm.

E) Western blot images showing protein expression levels of E-cadherin, vimentin, slug and GAPDH in total protein lysates taken from HaCaT cells without (Untreated) or with (+TGF-β & EGF) growth factor treatment for 5 days.

F) Representative time-lapse series of actin pattern development in HaCaT cells replated on 1800 μm^2 fibronectin-coated islands after 4 days of growth factor treatment. Cells were transfected with Lifeact-GFP one day before replating and imaging overnight. Time in hours after seeding is indicated in upper left corner of each frame. Scale bar: 10 μm.

Journal of Cell Science • Supplementary information

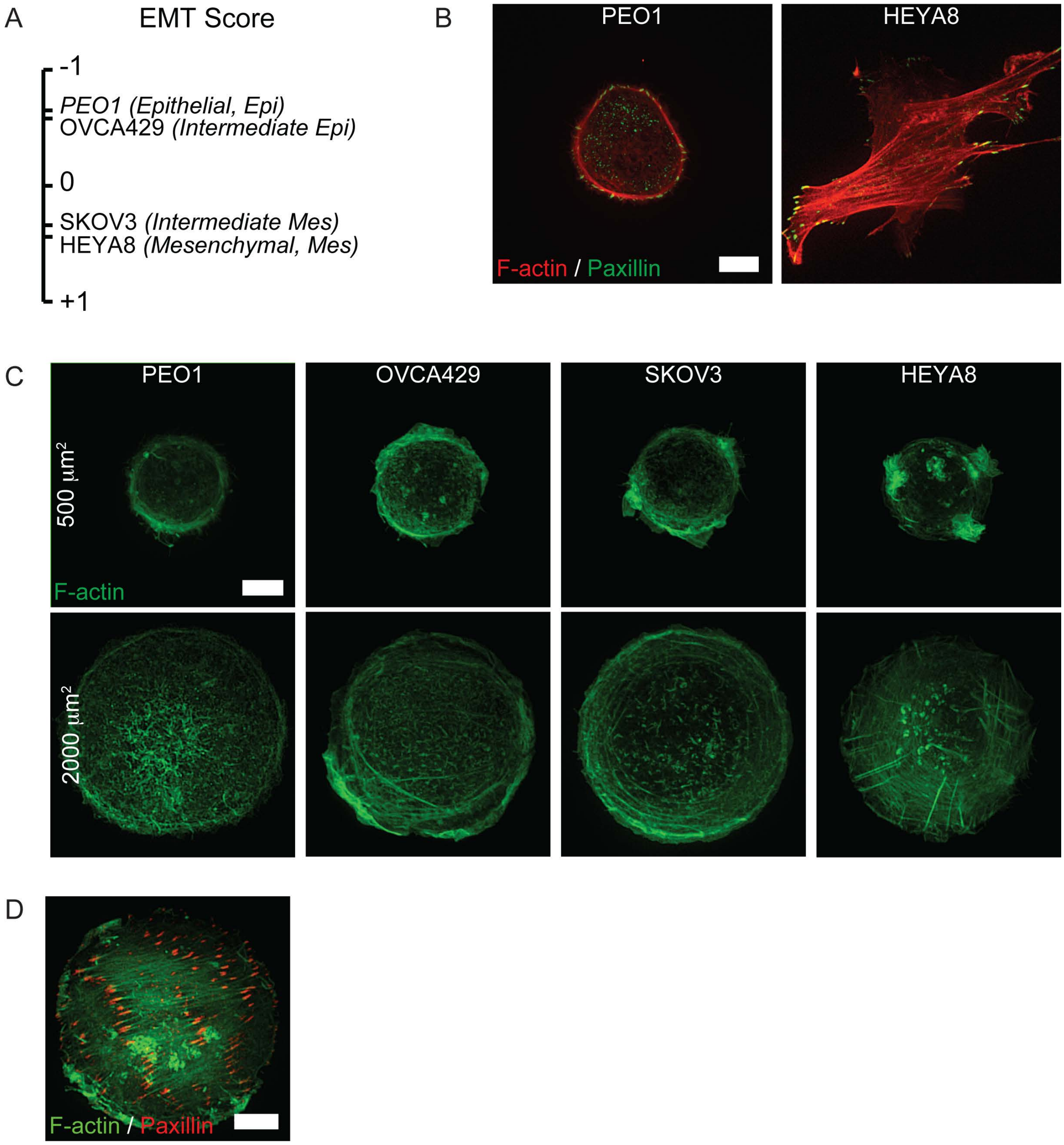
A
EMT Score
-1
PEO1 (Epithelial, Epi)
OVCA429 (Intermediate Epi)
0
SKOV3 (Intermediate Mes)
HEYA8 (Mesenchymal, Mes)
+1
B
PEO1
HEYA8
F-actin / Paxillin
C
PEO1
OVCA429
SKOV3
HEYA8
500 μm²
2000 μm²
F-actin
D
F-actin / Paxillin

Figure S4: Actin cytoskeleton self-organization in ovarian cancer cell lines

A) A selection of ovarian cancer cell lines (PEO1, OVCA429, SKOV3 and HEYA8) distributed along a scale corresponding to their EMT score (from most epithelial, -1 to most mesenchymal, +1).

B) Representative images showing focal adhesion (paxillin immunostaining) and F-actin (phalloidin staining) distribution in epithelial (PEO1) and mesenchymal (HEYA8) ovarian cancer cells fixed 16 h after seeding on planar fibronectin-coated substrates. Scale bar: 10 μm.

C) Representative images of ovarian cancer cells with different EMT phenotypes (PEO1, OVCA429, SKOV3 and HEYA8) fixed and stained with phalloidin 16 h after seeding on fibronectin-coated islands of different sizes. Scale bar: 10 μm.

D) Representative image showing focal adhesion (paxillin immunostaining) and F-actin (phalloidin staining) distribution in a HEYA8 cell imaged under conditions detailed in **C** that shows the linear actin pattern. Scale bar: 10 μm.

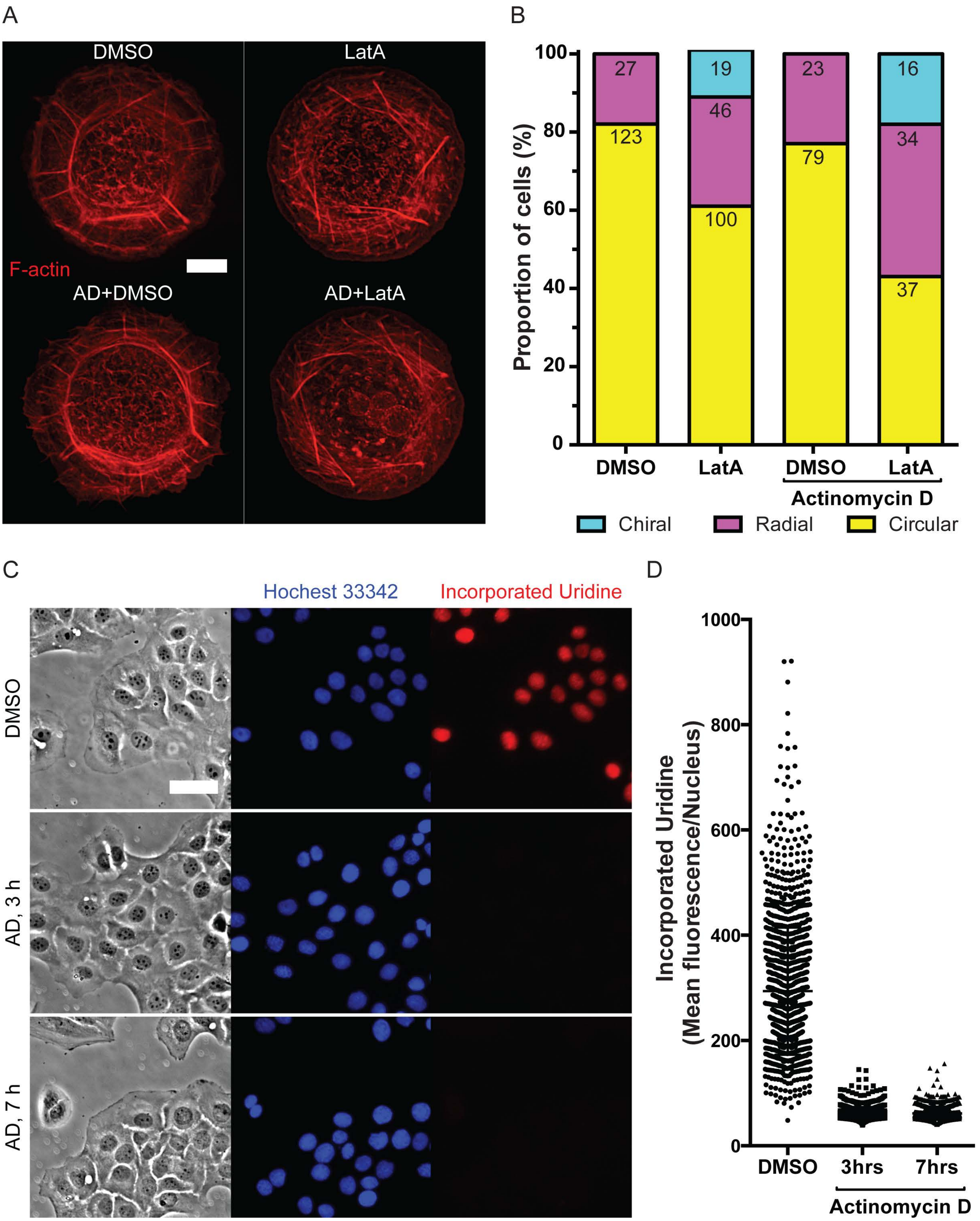
A
DMSO
LatA
F-actin
AD+DMSO
AD+LatA
B
Proportion of cells (%)
100
80
60
40
20
0
27
123
19
46
100
23
79
16
34
37
DMSO
LatA
DMSO
LatA
Actinomycin D
Chiral
Radial
Circular
C
Hochest 33342
Incorporated Uridine
DMSO
AD, 3 h
AD, 7 h
D
Incorporated Uridine
(Mean fluorescence/Nucleus)
1000
800
600
400
200
0
DMSO
3hrs
7hrs
Actinomycin D

Figure S5: Chiral actin swirling persists in latrunculin A-treated keratinocytes even when transcriptional changes are suppressed

A) Representative image showing F-actin distribution (phalloidin staining) in keratinocytes that were treated with actinomycin D (AD) or an equivalent volume of DMSO after seeding on 1800 μm^2 fibronectin-coated adhesive islands for 2 h and then incubated with actinomycin D during latrunculin A (LatA, 100 nM) treatment before fixing ~7 h after seeding. Scale bar: 10 μm.

B) Bar chart showing fractions of cells with actin cytoskeleton demonstrating circular, radial or chiral pattern from cells imaged under conditions of **A**. Numbers of cells displaying each actin pattern are labelled on top of each bar per condition, pooled from 2 independent experiments. Fisher's exact tests were used to assess significance between the fractions of cells displaying each actin pattern in pairs (DMSO vs. AD+DMSO, LatA vs. AD+LatA); no statistically significant differences were calculated.

C) Representative images showing brightfield images together with Hoechst 33342 and uridine staining in keratinocytes that were treated with DMSO (upper panel) or 2 $\mu g\ ml^{-1}$ actinomycin D for 3 h (middle panel) and 7 h (lower panel) after seeding on fibronectin-coated glass coverslips. Note actinomycin D treated cells lack uridine staining. Scale bar: 50 μm.

D) Scatter dot plot with Mean $\pm$ Standard Deviation showing the incorporation of uridine into newly transcribed RNA, measured from keratinocytes imaged under conditions of **C**.

Journal of Cell Science • Supplementary information

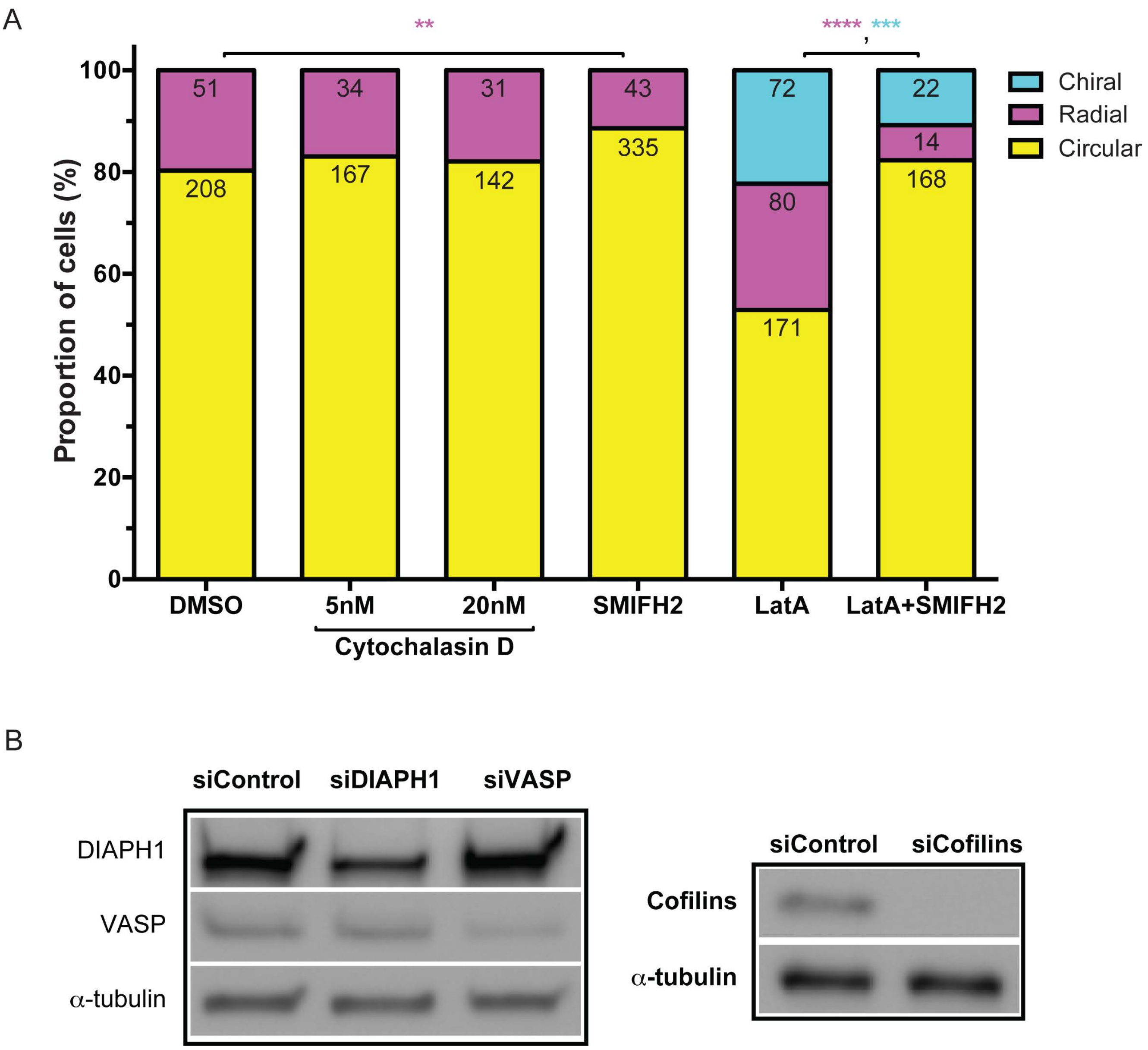

Journal of Cell Science • Supplementary information

Figure S6: Effect of inhibitors of actin polymerization on the formation of radial fibres and development of chiral actin patterns in keratinocytes

A) Bar chart showing fractions of cells with actin cytoskeleton demonstrating circular, radial or chiral pattern from HaCaT cells treated with DMSO, 5 nM cytochalasin D, 20 nM cytochalasin D, 15 μM SMIFH2, 100 nM latrunculin A (LatA), or 100 nM latrunculin A combined with 15 μM SMIFH2 (LatA+SMIFH2) and fixed after overnight seeding on 1800 μm^2 fibronectin-coated islands. Numbers of cells displaying each actin pattern are labelled on top of each bar per condition, pooled from 2 independent experiments. Fisher's exact tests were used to assess significance between the fractions of cells displaying radial (magenta asterisk) or chiral (cyan asterisk) actin pattern in pairs (DMSO vs. 5 nM Cytochalasin D, DMSO vs. 20 nM Cytochalasin D, DMSO vs. SMIFH2, LatA vs. LatA+SMIFH2); *P<0.05, **P<0.01, ***P<0.001, ****P<0.0001.

B) Western blot images showing protein expression levels of DIAPH1, VASP, Cofilins (1 and 2 together) and α-tubulin in total protein lysates taken from HaCaT cells genetically silenced with Control, DIAPH1, VASP and Cofilins (1 and 2 together) siRNAs.

Journal of Cell Science • Supplementary information

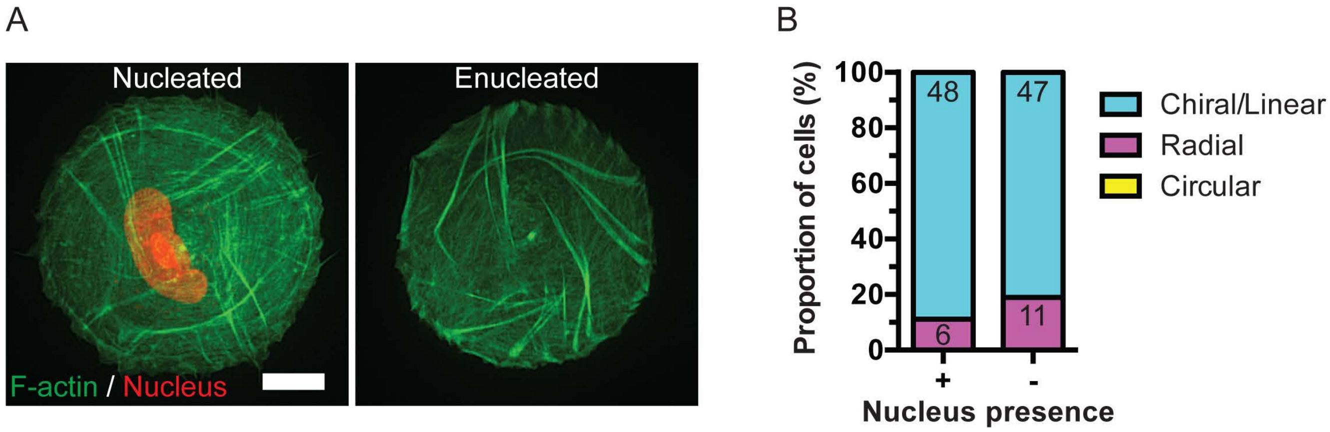

Figure S7: Chiral actin swirling persists in enucleated fibroblasts

A) Representative frames showing actin cytoskeleton development in nucleated and enucleated (see Materials and methods) HFF cells plated on 1800 μm^2 fibronectin-coated adhesive islands, expressing GFP-Lifeact with nucleus marker (BFP-NLS) and additionally stained by Hoechst 33342. Scale bar: 10 μm.

B) Bar chart showing fractions of cells with actin cytoskeleton demonstrating circular, radial or chiral pattern from cells imaged under conditions of **A**. Numbers of cells displaying each actin pattern are labelled on top of each bar per condition, pooled from 2 independent experiments. Fisher's exact test was used to assess the significance between the fractions of cells displaying the radial or chiral actin pattern; no statistically significant difference was calculated.

Journal of Cell Science • Supplementary information

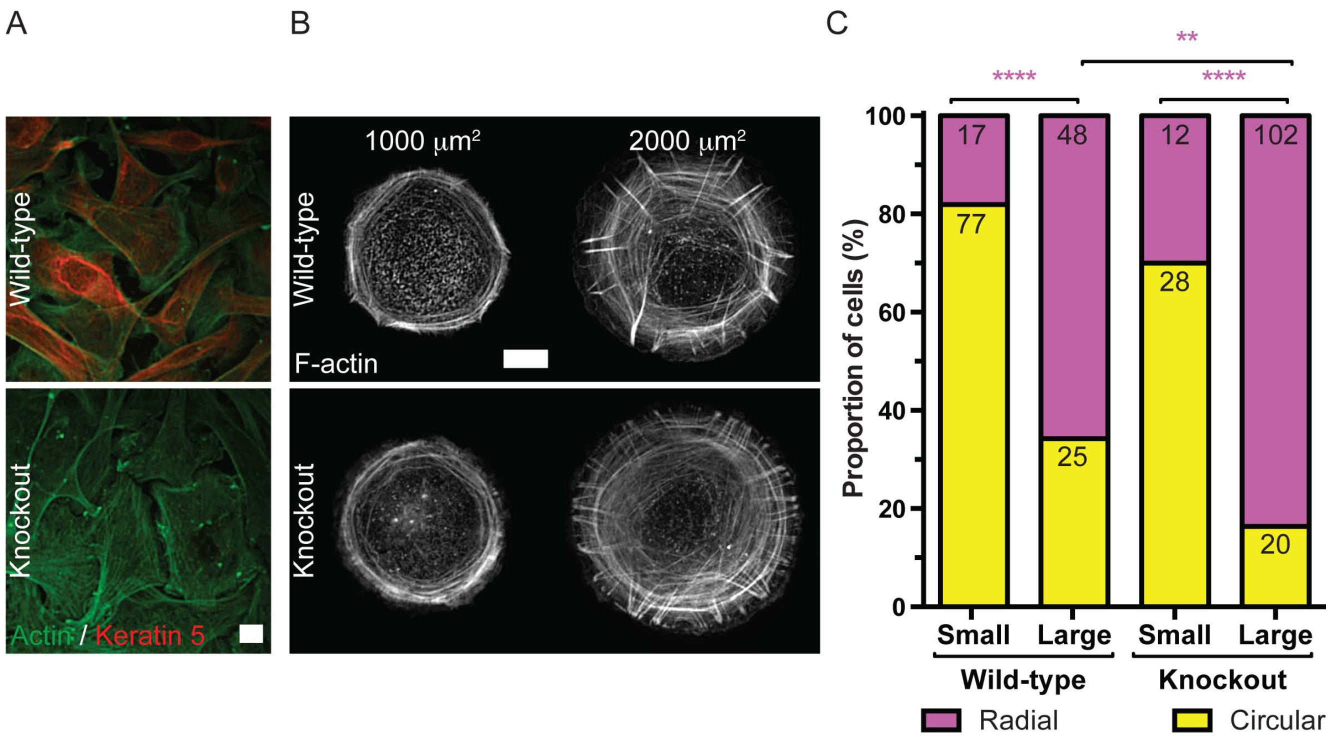

Figure S8: Comparison of actin cytoskeleton self-organization between wild-type and keratin-null murine keratinocytes

A) Representative images showing Actin (β-actin immunostaining) and Keratin5 (cytokeratin5 immunostaining) distribution in murine keratinocytes with normal (Wild-type) or depleted (Knockout) cytokeratin expression fixed after seeding on collagen-coated coverslips. Note Keratin5 staining was absent in Knockout cells. Scale bar: 10 μm.

B) Representative images showing actin patterns in murine keratinocytes with normal (Wild-type) or depleted (Knockout) cytokeratin expression. Images show F-actin distribution (phalloidin staining) 24 h after seeding on collagen-coated islands of different sizes. Scale bar: 10 μm.

C) Bar chart showing fractions of cells with actin cytoskeleton demonstrating circular or radial pattern from Wild-type or Knockout murine keratinocytes plated on Small (500, 750, 1000 μm^2) or Large (1500, 2000, 2500 μm^2) collagen-coated adhesive islands. Numbers of cells displaying each actin pattern are labelled on top of each bar, pooled from 3 and 2 independent experiments for Wild-type and Knockout cells respectively. Fisher's exact tests were used to assess significance between the fractions of cells displaying radial actin pattern in pairs (Wild-type on Small vs. Wild-type on Large, Wild-type on Small vs. Knockout on Small, Wild-type on Large vs. Knockout on Large, Knockout on Small vs. Knockout on Large); *P<0.05, **P<0.01, ***P<0.001, ****P<0.0001.

Journal of Cell Science • Supplementary information

Supplemental Tables

Table S1: Non-keratinocyte epithelial cell spreading capacity on adhesive islands

Table showing the number of cells under conditions shown in Fig. 2 that filled the circular shape when seeded on adhesive islands of different sizes (500, 750, 1000, 1500, 2000, 2500 μm^2). The number of cells selected after examination of 4, 3 and 2 dishes for MCF-10A, MCF-7 and NBT-II respectively are indicated.

**Cell line**	**MCF-10A**	**MCF-7**	**NBT-II**
**Number of independent experiments**	4	3	2
**Projected cell area (μm^2)**	**Number of cells filling adhesive islands**		
**500**	34	12	15
**750**	9	9	8
**1000**	5	4	10
**1500**	0*	1	5
**2000**	0	0	1
**2500**	0	0	1

*no cells filled adhesive islands of this size

Table S2: Ovarian cancer cell lines spreading capacity on adhesive islands

Table showing the number of cells under conditions shown in Fig. S4C that filled the circular shape when seeded on fibronectin-coated adhesive islands of different sizes (500, 750, 1000, 1500, 2000, 2500 μm^2).

**Cell line**	**PEO1**	**OVCA429**	**SKOV3**	**HEYA8**
**Projected cell area (μm^2)**	**Number of cells filling adhesive islands**			
**500**	23	16	3	3
**750**	11	8	10	7
**1000**	4	18	13	13
**1500**	0*	11	18	12
**2000**	2	13	19	15
**2500**	0	2	10	12

*no cells filled adhesive islands of this size

Journal of Cell Science • Supplementary information

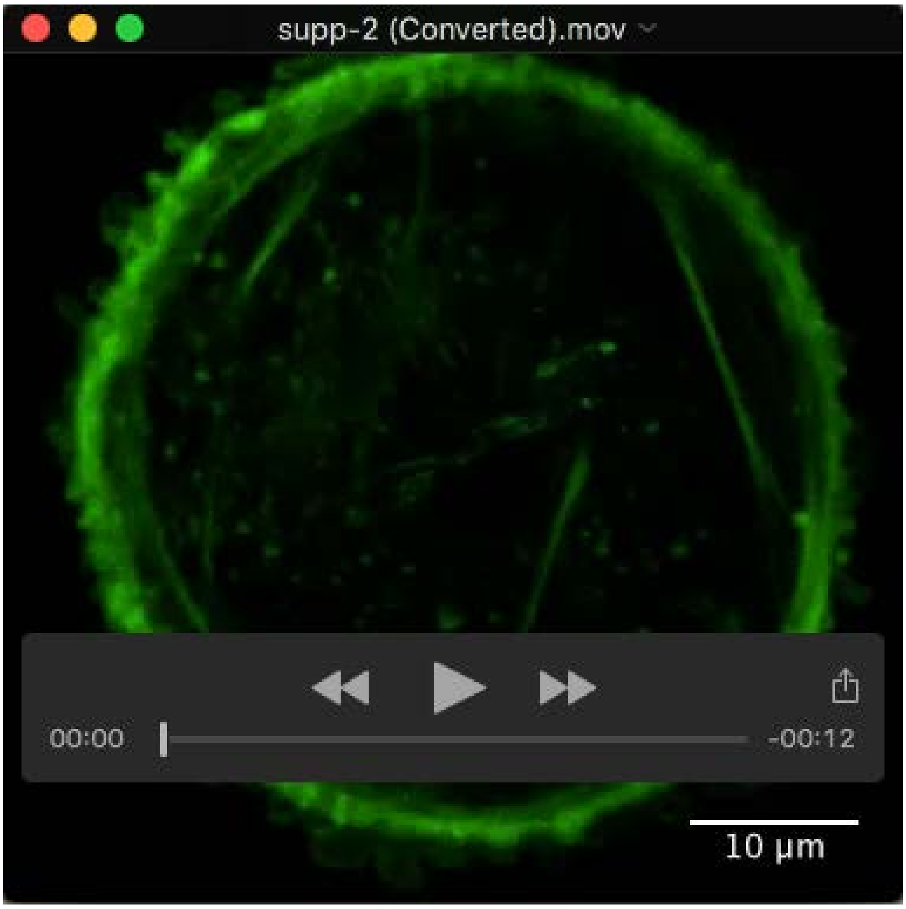

Movie 1: Evolution of actin cytoskeleton self-organization in fibroblasts

Representative time-lapse series showing actin cytoskeleton development in HFF cells expressing Lifeact-GFP plated on an 1800 μm^2 adhesive fibronectin-coated island. Note ventral stress fibres from first frame remain stable for first hour of movie. Scale bar: 10 µm. Timestamp: hh:mm. Playback rate: 10 frames sec^{-1}.

Journal of Cell Science • Supplementary information

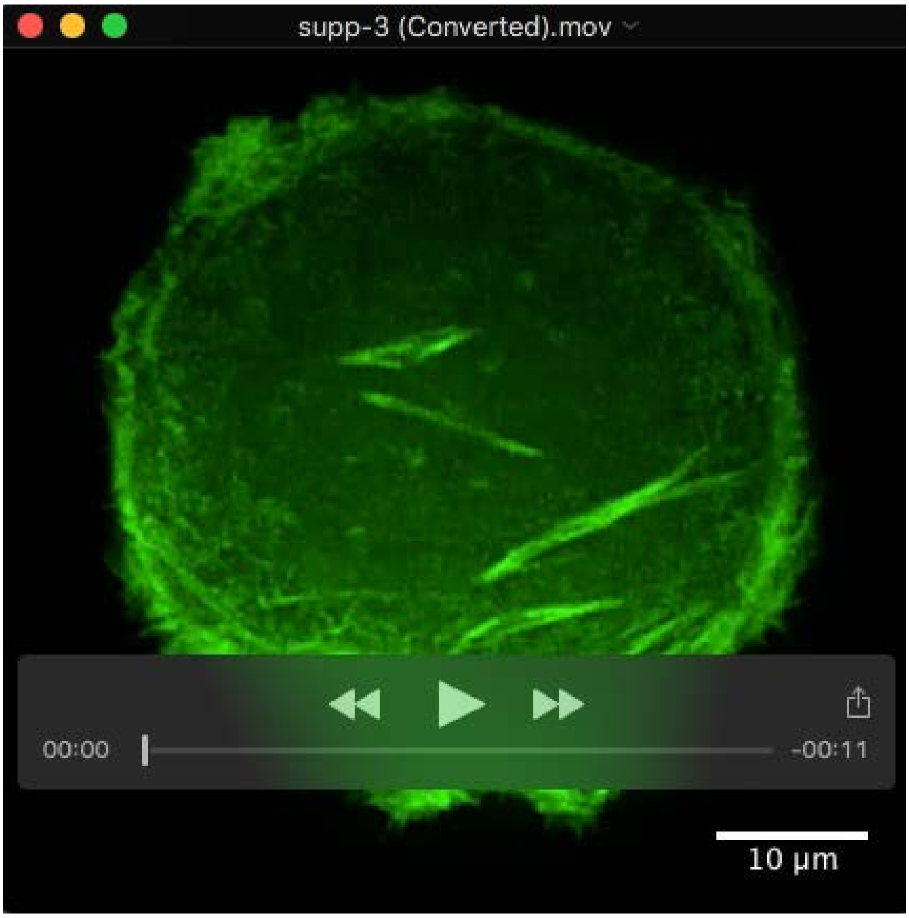

Movie 2: Evolution of actin cytoskeleton self-organization in non-keratinocyte epithelial cells

Representative time-lapse series showing actin cytoskeleton development in NBT-II cells expressing Lifeact-GFP plated on an 1800 μm^2 adhesive fibronectin-coated island. Note appearance of new short ventral stress fibres in the bottom half of cell over the first hour of movie. Scale bar: 10 µm. Timestamp: hh:mm. Playback rate: 20 frames sec^{-1}.

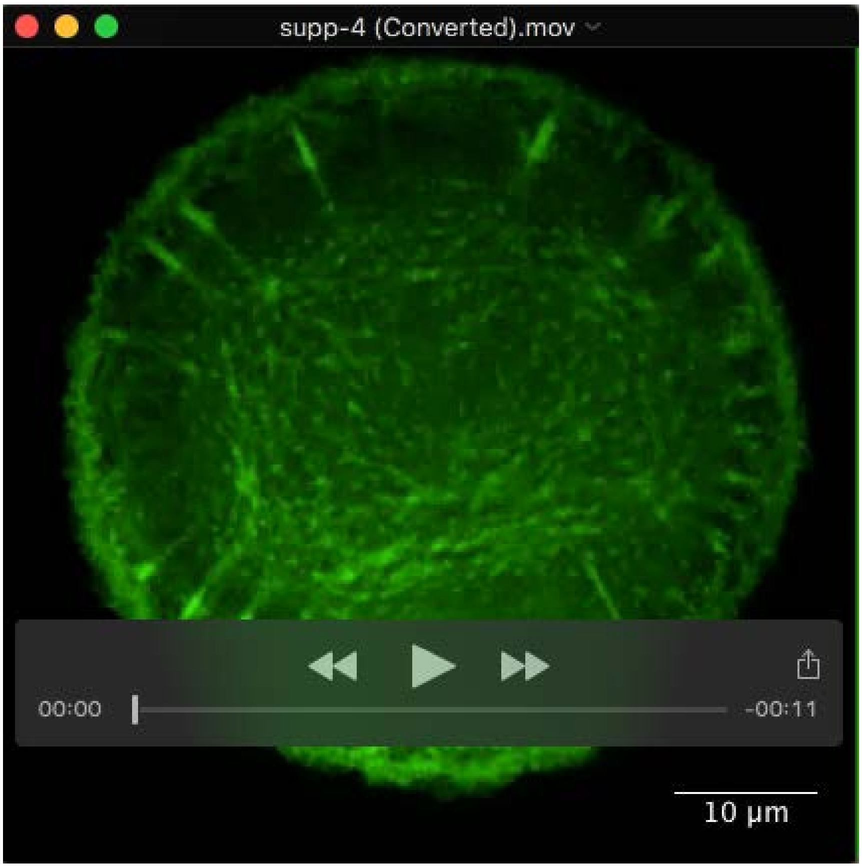

Movie 3: Evolution of actin cytoskeleton self-organization in non-keratinocyte epithelial cells after epithelial-mesenchymal transition

Representative time-lapse series showing actin cytoskeleton development in NBT-II cells expressing Lifeact-GFP pretreated with 100 ng ml^{-1} Epidermal Growth Factor (EGF) for 3 days and replated on 1800 μm^2 fibronectin-coated islands in the continued presence of EGF. Scale bar: 10 µm. Timestamp: hh:mm. Playback rate: 20 frames sec^{-1}.

Journal of Cell Science • Supplementary information

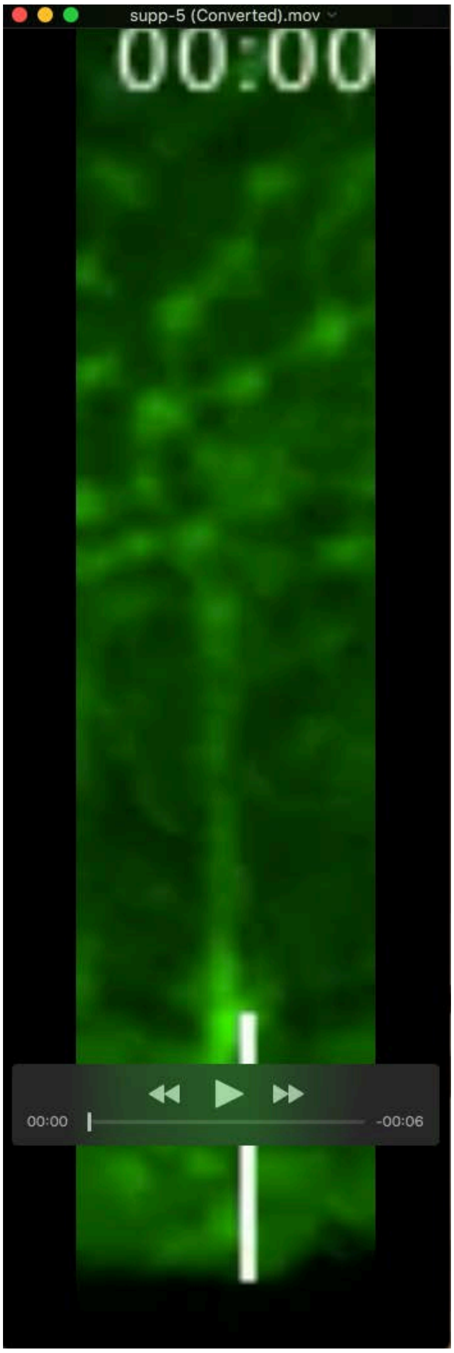

Movie 4: Centripetal directed movement of polygonal actin networks in EGF treated NBT-II cells

One hour long time-lapse showing region of actin cytoskeleton development in NBT-II cells expressing Lifeact-GFP pretreated with 100 ng ml^{-1} Epidermal Growth Factor (EGF) for 3 days and replated on 1800 µm^2 fibronectin-coated islands in the continued presence of EGF, taken from region highlighted by a white box shown in the first frame of the EGF treated cell from Fig. 3A. Scale bar: 5 µm. Timestamp: hh:mm. Playback rate: 5 frames sec^{-1}.

Journal of Cell Science • Supplementary information

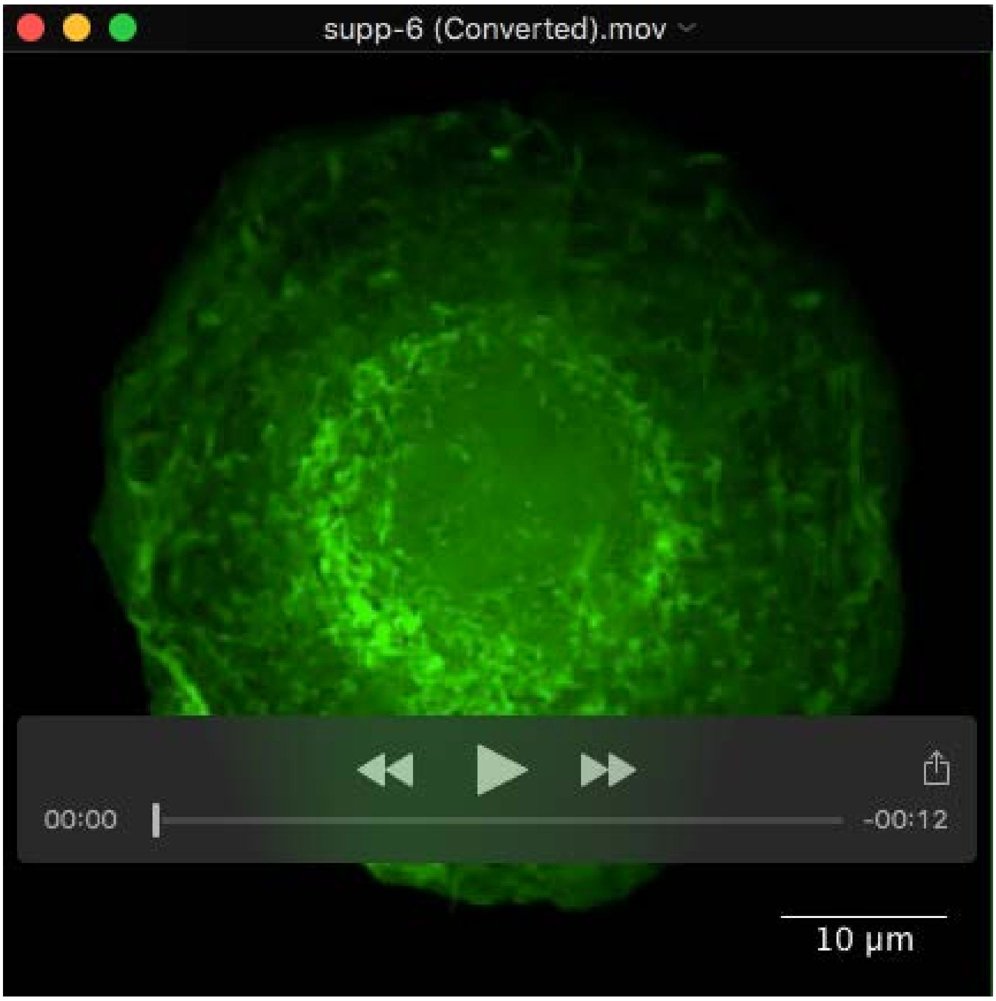

Movie 5: Evolution of actin cytoskeleton self-organization in keratinocytes

Representative time-lapse series showing actin cytoskeleton development in HaCaT cells expressing Lifeact-GFP plated on an 1800 μm^2 adhesive fibronectin-coated island. Scale bar: 10 µm. Timestamp: hh:mm. Playback rate: 10 frames sec^{-1}.

Journal of Cell Science • Supplementary information

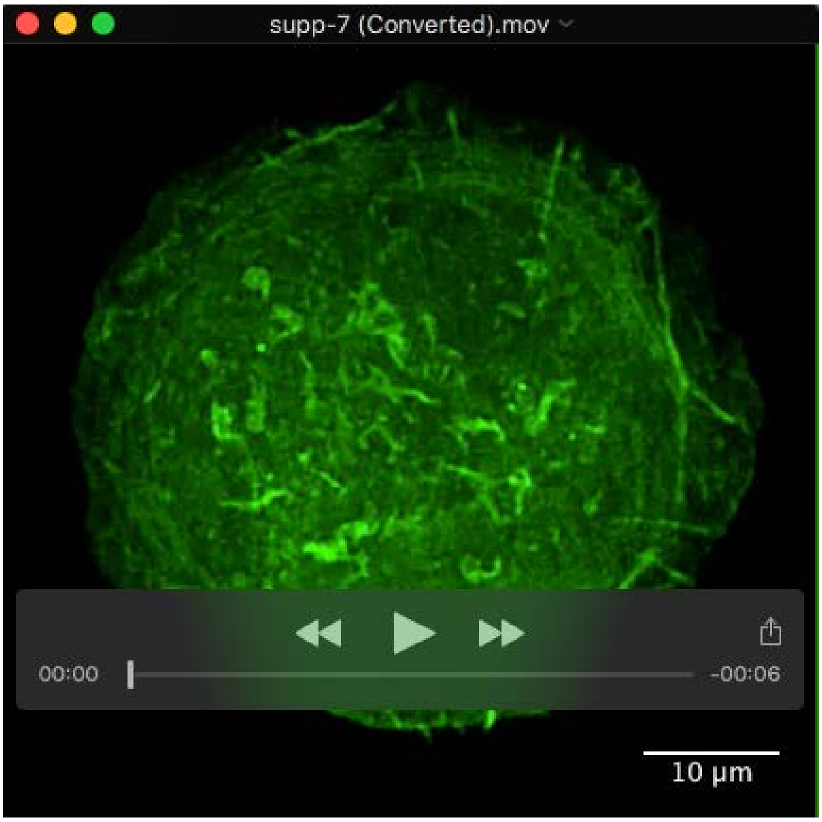

Movie 6: Induction of chiral actin pattern development in keratinocytes

Representative time-lapse series showing actin cytoskeleton development in HaCaT cells expressing Lifeact-GFP plated on an 1800 µm^2 adhesive fibronectin-coated island treated with 20 nM latrunculin A. Latrunculin A was added to cells 8 h after seeding. Scale bar: 10 µm. Timestamp: hh:mm. Playback rate: 10 frames sec^{-1}.

Journal of Cell Science • Supplementary information

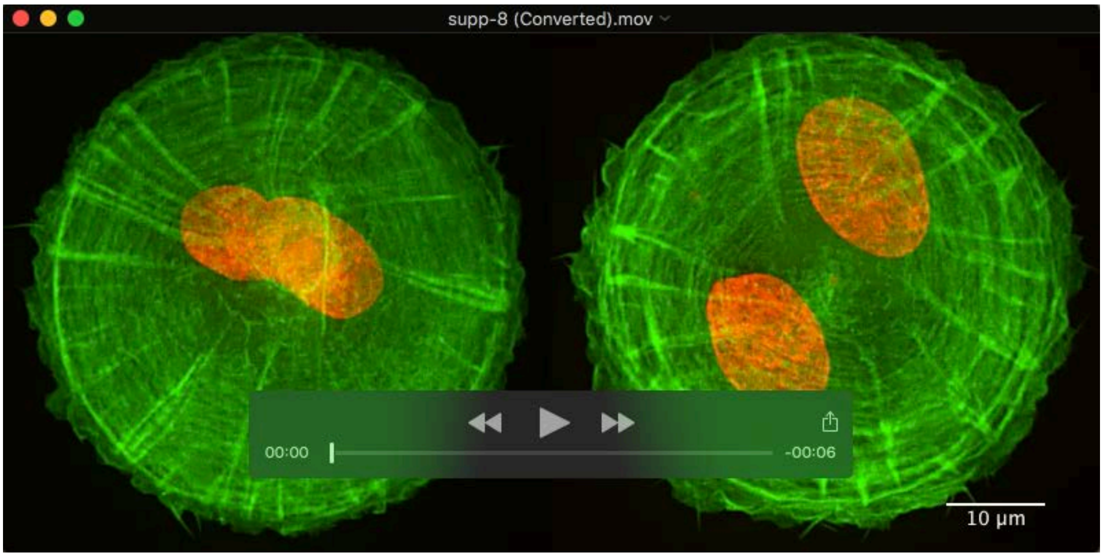

Movie 7:

Representative time-lapse series showing actin cytoskeleton development and nucleus rotation in diploid (left, untreated cells) or tetraploid (right, achieved by pre-treatment with cytochalasin D) HFF cells expressing GFP-Lifeact with BFP-NLS seeded on an 1800 μm^2 fibronectin-coated island. Scale bar: 10 μm. Timestamp: hh:mm. Playback rate: 10 frames sec^{-1}.

Journal of Cell Science • Supplementary information

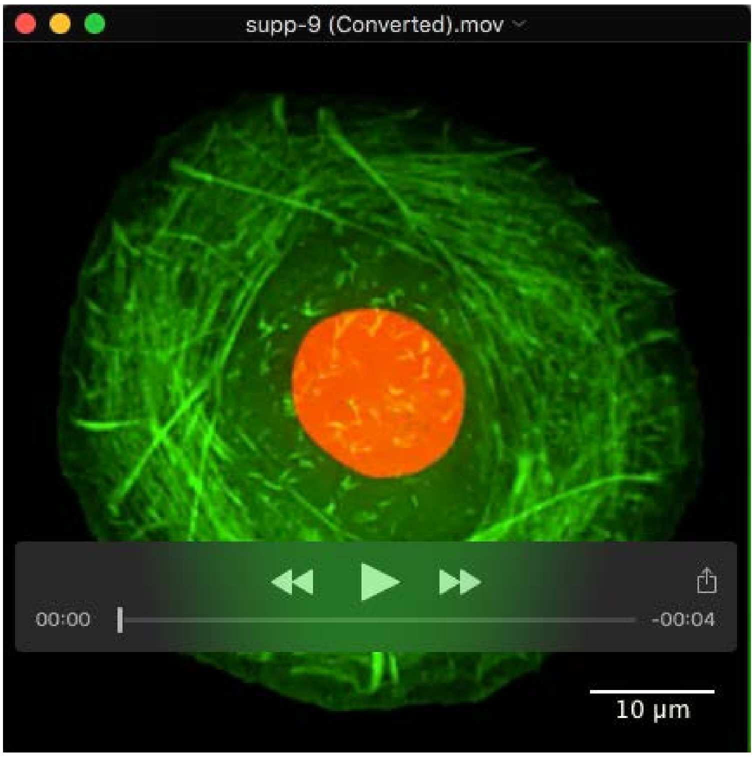

Movie 8: Rotation of the nucleus in latrunculin A-treated keratinocytes

Representative time-lapse series showing actin cytoskeleton development and nucleus rotation in latrunculin A-treated HaCaT cells expressing GFP-Lifeact with RFP-H1 seeded on a 2000 μm^2 fibronectin-coated island. 200 nM of latrunculin A was added to cells 40 minutes after seeding. Scale bar: 10 µm. Timestamp: hh:mm. Playback rate: 10 frames sec^{-1}.

Journal of Cell Science • Supplementary information

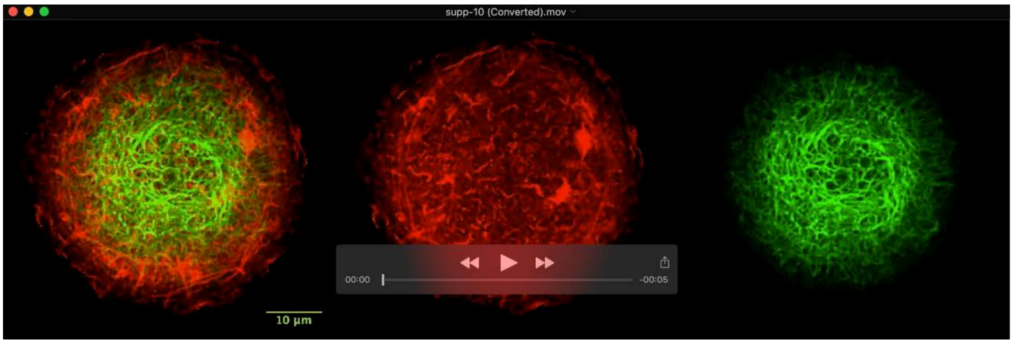

Movie 9: Evolution of actin and keratin cytoskeleton self-organization in keratinocytes

Representative time-lapse series showing actin and keratin cytoskeleton development in HaCaT cells expressing mRuby-Lifeact and mEmerald-Keratin14 plated on an 1800 μm^2 adhesive fibronectin-coated island. Scale bar: 10 µm. Timestamp: hh:mm. Playback rate: 10 frames sec^{-1}.

Journal of Cell Science • Supplementary information

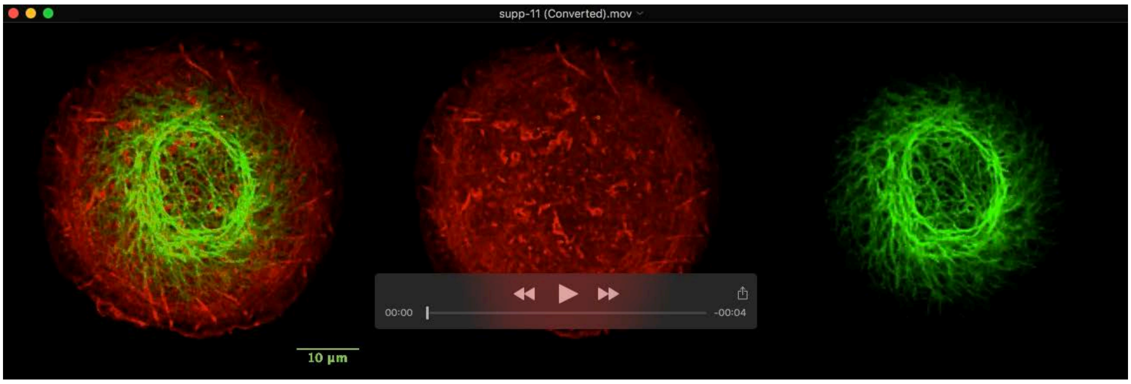

Movie 10: Evolution of actin and keratin cytoskeleton self-organization in keratinocytes in the chiral actin pattern

Representative time-lapse series showing cytoskeleton progression in latrunculin A-treated HaCaT cells expressing mEmerald-Keratin14 with mRuby-Lifeact seeded on 1800 μm^2 fibronectin-coated islands. 20 nM of latrunculin A was added to cells 8 h after seeding. Scale bar: 10 µm. Timestamp: hh:mm. Playback rate: 10 frames sec^{-1}.

Journal of Cell Science • Supplementary information
